# A self-assembled macrocycle with a non-closed structure for hierarchically upgraded self-assembly

**DOI:** 10.1039/d5sc06544e

**Published:** 2025-11-11

**Authors:** Ze Cao, Chenqi Ge, Guangcheng Wu, Hua Tang, Yating Wu, Yueyan Kuang, Yuyang Lu, Jiyong Liu, Hao Li

**Affiliations:** a Departmsent of Chemistry, Zhejiang University Hangzhou 310058 China; b Department of Chemistry, The University of Hong Kong Hong Kong SAR 999077 China; c ZJU-Hangzhou Global Scientific and Technological Innovation Center Hangzhou 311215 Zhejiang Province China

## Abstract

Here, a [3 + 10] non-closed macrocycle was serendipitously self-assembled, incorporating two monovalent diamines, each with an unreacted amino group. These are selectively removed *via* imine exchange, yielding a dialdehyde intermediate. This precursor facilitates hierarchical self-assembly into structures comprising two types of amine building blocks unattainable by one-pot synthesis.

## Abstract 2

A [3 + 10] non-closed macrocycle was self-assembled, which facilitates hierarchical self-assembly into structures with different amine building blocks unattainable by one-pot synthesis.

## Graphical abstract

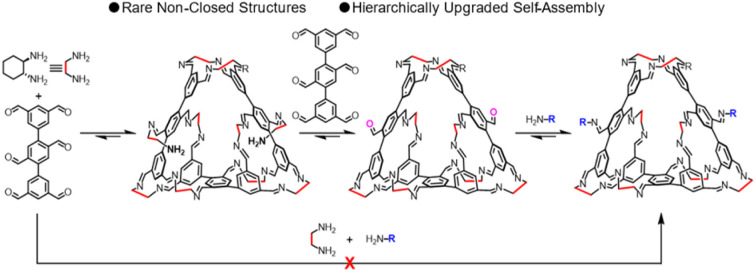

## Introduction

**Fig. 1:**
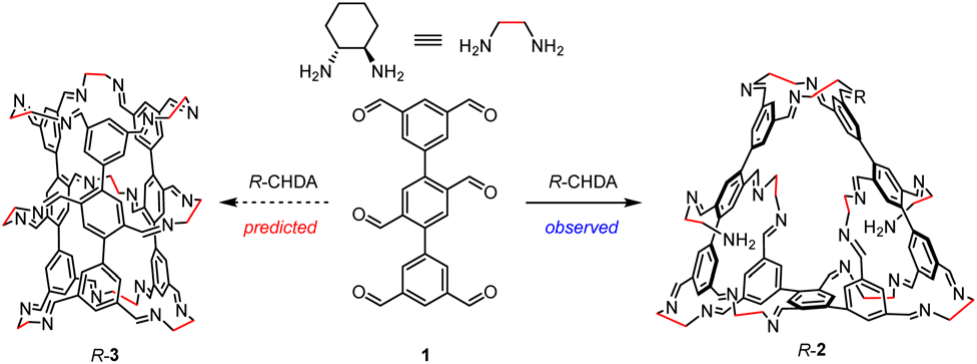
Structural formulae of the predicted [3 + 9] tube *R*-3 and the experimentally observed [3 + 10] non-closed macrocycle *R*-2 by performing imine formation reaction to a mixture of hexaformyl precursor 1 and *R*-CHDA.

Self-assembly based on dynamic bonds,^1–11^ such as imine bonds,^12–23^ represents a more advanced approach compared to traditional methods relying on irreversible organic reactions. This approach allows the system to undergo error correction, leading to the formation of the most thermodynamically favored products in a one-pot manner. This advantage is particularly prominent for molecules comprising numerous components, as their synthesis requires forming multiple bonds and would otherwise be technically challenging using irreversible reactions. However, because self-assembled products typically occupy thermodynamic minima, the system tends to maximize bond formation. Consequently, molecules with open architectures that retain unreacted ligation sites are inherently difficult to synthesize *via* dynamic approaches. Such self-assembled products typically cannot serve as precursors for higher-order self-assembly. Here, we condensed a hexaformyl precursor with a chiral diamine in chloroform. Instead of forming the anticipated tube-shaped molecule with a closed architecture, a macrocycle with an open structure was self-assembled as the sole observable product (Fig. 1). This macrocycle comprises three equivalents of the hexaformyl precursor and ten equivalents of the diamine precursor. Notably, two of the ten diamine residues each retain one unreacted amino group and are connected to the macrocycle *via* only a single imine bond. Unlike other eight diamine units that form two imine linkages, these two units can be readily removed through imine exchange or hydrolysis without disrupting the macrocycle's integrity. Consequently, the resulting product serves as a diformyl precursor suitable for hierarchically upgraded self-assembly by reacting with various amines, enabling the construction of a variety of structures containing two types of amine building blocks that are difficult or unlikely to synthesize *via* one-pot approaches.

Our study originated from a serendipitous discovery. Condensing *m*-phthalaldehyde or *p*-phthalaldehyde with one enantiomer of *trans*-1,2-cyclohexanediamine (namely their *R*- or *S*-CHDA) was shown^24–27^ to self-assemble a triangular macrocycle. This macrocycle comprises three equivalents of the dialdehyde and three equivalents of *trans*-CHDA and is formed as the sole observable product in near-quantitative yield. This triangular product represents the thermodynamically favored structure because the two amino groups in CHDA define a 60° angle within a plane, enabling an optimal geometry for triangle formation. Subsequent work by our group^28,29^ and others,^21,30–37^ including Cooper and co-workers,^38–40^ demonstrated that various molecular tubes could self-assemble. Each tube can be considered as a “dimer” of triangles, formed by condensing three equivalents of a tetraformyl precursor (containing two *m*-phthalaldehyde units grafted onto an aromatic core) with six equivalents of *trans*-CHDA. Remarkably, this assembly remained efficient even with insoluble tetraformyl precursors, facilitated by CH–π interactions between the three central aromatic units. However, these CH–π interactions also cause the aromatic panels to stack closely, which leads to a collapse of the tube frameworks and thereby deprives them of effective cavities.^28,29^ Consequently, unlike the previously reported molecular tube systems containing pillararenes^41–46^ and resorcinarenes^47–51^ building blocks that endow pre-organized cavities, none of these self-assembled tubes composed of *trans*-CHDA precursors exhibit guest accommodation ability. We therefore propose that introducing two additional formyl units onto the central aromatic spacer of the tetraformyl precursor would lead to the formation of a third triangular macrocycle in the central part of the molecular tube, thus creating a pre-organized cavity for effective guest recognition.

## Results and discussion

The hexaformyl precursor 1 was synthesized *via* Suzuki coupling of a 1 : 2.4 mixture of 2,5-dibromo-*p*-phthalaldehyde and an *m*-phthalaldehyde derivative bearing a boronic ester (Scheme S1, SI). Due to its insolubility in almost all organic solvents, 1 was precipitated directly from the Suzuki reaction mixture and used for self-assembly without further purification or characterization. We suspended crude compound 1 (199 mg, 0.5 mmol, unpurified) in CHCl_3_ (500 mL), then added *R*-CHDA (228 mg, 2.0 mmol) and a catalytic amount of CF_3_COOH (50 µL). The mixture was stirred at room temperature for 48 hours. During this period, most solid 1 was dissolved, leaving a small amount of insoluble material (likely Suzuki reaction impurities), which was removed by filtration. The clear filtrate was concentrated, after which MeOH was added to precipitate the self-assembly product *R*-2 as a white powder (94 mg). *R*-2 exhibited good solubility in most non-polar solvents. The ^1^H NMR spectrum recorded in CDCl_3_ showed a single set of well-defined resonances corresponding to a pure species, as confirmed by DOSY spectrum (Fig. S6, SI). The isolated yield of *R*-2 was 31% over two steps, relative to the dibromo-*p*-phthalaldehyde precursor used in the Suzuki reaction. Compound *S*-2 was synthesized analogously using *S*-CHDA, exhibiting mirror-image circular dichroism (CD) signals compared to *R*-2 (Fig. S34, SI). Notably, when 1 was combined with racemic *trans*-CHDA, only an ill-defined mixture of products was obtained (Fig. S12, SI). This indicates that homochiral products (*R*-2 nor *S*-2) are not sufficiently thermodynamically favored to drive narcissistic self-sorting, unlike the behavior observed in reported molecular tube systems.^40^

**Fig. 2:**
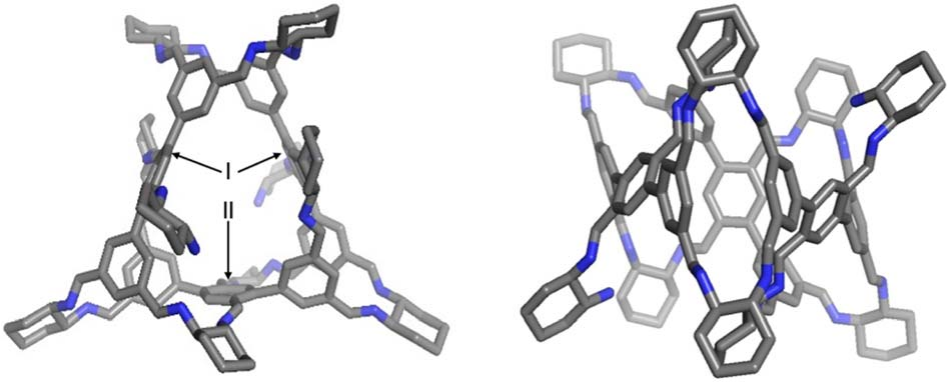
Side and top views of the solid-state structure of *R*-2, which were obtained *via* single crystal X-ray diffraction analysis. I and II represent distinct precursor positions. Carbon, grey; nitrogen, blue. Hydrogen atoms and solvent molecules are removed for clarity.

Diffraction-grade single crystals of *R*-2 were obtained by slow diffusion of methyl *tert*-butyl ether into its chloroform solution, unambiguously confirming its structure (Fig. 2). In the solid state, *R*-2 comprises ten *R*-CHDA residues and three equivalents of precursor 1, consistent with molecular ion peaks observed in high-resolution mass spectrometry (Fig. S7). To minimize steric hindrance, all imine protons adopt a *syn* conformation relative to the corresponding methine protons in the *R*-CHDA residues, as observed in other reported systems with CHDA building blocks.^20^ Within the framework of *R*-2, the three residues of precursor 1 occupy two distinct positions: the two hexaformyl precursors in position I are interconnected *via* two *R*-CHDA bridges, whereas three additional *R*-CHDA bridges connect the hexaformyl building blocks in positions I and II (Fig. 2). In the two precursors located in position I, the central *p*-phthalaldehyde unit contains a formyl group connected to an *R*-CHDA residue bearing an unreacted amino group. This connectivity imparts a *C*_2_ rotational symmetry axis to *R*-2.

**Fig. 3:**
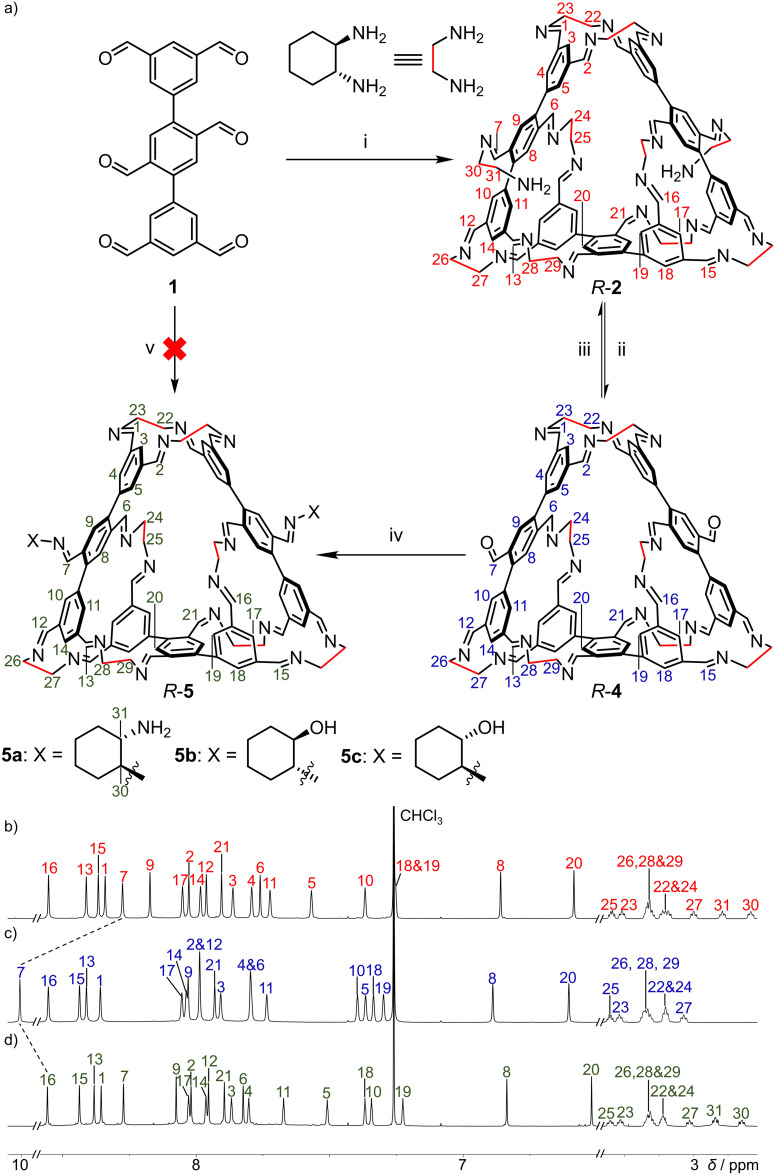
(a) Structural formulae of *R*-2, *R*-4 and *R*-5, each containing three equivalents of hexaformyl precursor 1, all of these three structures exhibit *C*_2_ symmetry. (i) *R*-CHDA, CHCl_3_, 24 h at r.t., 31% in two steps relative to 2,5-dibromoterephthalaldehyde; (ii) 1, CHCl_3_, 48 h at r.t., 92%; (iii) *R*-CHDA, CDCl_3_, 24 h at r.t., 93%; (iv) 6 = X–NH_2_, CDCl_3_, 24 h at r.t., 72%, 85% and 79% for *R*-5a, *R*-5b and *R*-5c; (v) *R*-CHDA and 6, CDCl_3_, not obtained. Partial ^1^H NMR (600 MHz, CDCl_3_, 298 K) spectra of (b) *R*-2, (c) *R*-4 and (d) *R*-5a.

The solid-state single-crystal structure of *R*-2 fully aligns with its solution-phase ^1^H NMR spectra. Signal assignments were confirmed by two-dimensional NMR techniques (Fig. S3–S5, SI). Twenty-one resonances were observed in the downfield region (*δ* 9.0–6.5 ppm) (Fig. 3b recorded in CDCl_3_, Fig. S1 recorded in CD_2_Cl_2_ and Fig. S10 recorded in C_2_D_2_Cl_4_, SI). This observation confirms the *C*_2_ rotational symmetry of *R*-2, as the structure contains forty-two equivalent sets of imine and phenyl protons. Among these aromatic protons, the resonances corresponding to protons 8 and 20 exhibit significant upfield shifts, indicating magnetic shielding effects (Fig. 3b). This finding correlates with the solid-state structure, where protons 8 and 20 are positioned above adjacent phenyl units. In the upfield region, ten methine proton resonances were observed, consistent with *R*-2 containing ten *R*-CHDA residues, each bearing two methine protons.

**Fig. 4:**
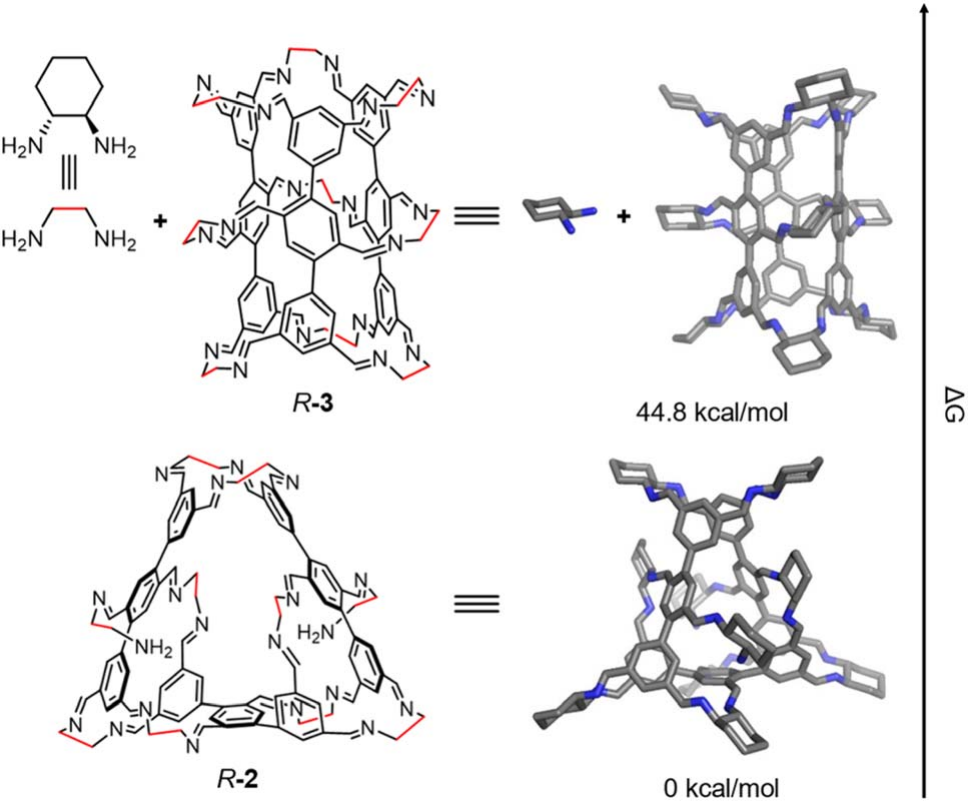
The Gibbs free energy differences (Δ*G*) between *R*-2 and *R*-3 + *R*-CHDA. Nitrogen, blue; carbon, grey. Hydrogen atoms and irrelevant solvent molecules are removed for clarity.

To elucidate the preferential formation of *R*-2 over the predicted tube-shaped structure *R*-3 during self-assembly, we performed density functional theory (DFT) calculations. While the hypothesized *R*-3 contains nine *R*-CHDA residues, *R*-2 incorporates one additional *R*-CHDA building block. Therefore, we compared the Gibbs free energy of *R*-2 with that of a binary mixture of *R*-3 and *R*-CHDA (determined by comparing the Δ*G* of the following two processes: the conversion of 3 eq. of the observed [1 + 6] intermediate into 1 eq. of *R*-2 and 8 eq. of *R*-CHDA or 1 eq. of *R*-3 and 9 eq. of *R*-CHDA, detailed in SI). DFT calculations revealed that *R*-2 is 44.8 kcal mol^−1^ lower in energy than the combined *R*-3 and *R*-CHDA system (Fig. 4). We also evaluated the energies of a few other possible self-assembly products, including *R*-3* (Fig. S36). *R*-3* is an analogue of *R*-3 where one central *R*-CHDA residue is replaced by two *R*-CHDA residues, each bearing an unreacted amino group. Its calculated Gibbs free energy was significantly higher than that of *R*-2, consistent with the experimental observation that *R*-2 is the predominant self-assembly product.

*R*-2 contains two peculiar *R*-CHDA residues, each connected to the macrocyclic moiety *via* only one imine linkage. It is thus predictable that these two residues are more susceptible to removal *via* imine exchange or hydrolysis compared to the other eight doubly connected diamine residues. We thus combined *R*-2 and 1 in a 1 : 5 ratio in CDCl_3_, the ^1^H NMR spectrum after 48 h at room temperature revealed a set of well-defined resonances corresponding to a new macrocycle, *R*-4, which features a non-closed structure. *R*-4 is the counterpart of *R*-2 formed by removing the two *R*-CHDA residues with single imine linkages *via* imine exchange. The formation of *R*-4 was fully characterized by ^1^H NMR spectroscopy and mass spectrometry (Fig. 3c and S21, SI). For instance, the ^1^H NMR spectrum of *R*-4 exhibited a characteristic resonance at *δ* 10.0 ppm corresponding to the formyl protons. *R*-4 could also be self-assembled directly by combining *R*-CHDA with excess 1 in CDCl_3_. Early in the time course of the reaction, a species characterized by five aromatic resonances in the ^1^H NMR spectrum was observed (Fig. S13, SI). Mass spectrometry indicated this species as a [1 + 6] product composed of one equivalent of 1 and six equivalents of *R*-CHDA (Fig. S14, SI). The formation of this [1 + 6] product is unsurprising, on account of the poor solubility of hexaformyl 1 in CDCl_3_, which maintains *R*-CHDA in excess relative to 1 in solution. This [1 + 6] product diminished within a few hours, while the [3 + 10] product *R*-2 became the dominant species. However, in the presence of excess hexaformyl precursor 1, *R*-2 was subsequently transformed into *R*-4 in the final stage of the self-assembly process (Fig. S22, SI).

The presence of two free formyl group in *R*-4 prompted its usage as a precursor for hierarchically upgraded self-assembly. Combining *R*-4 with excess *R*-CHDA regenerated *R*-2. When *R*-4 was combined with various other amine compounds, a variety of macrocyclic products each containing two types of amine building blocks were obtained, including *R*-5a, *R*-5b and *R*-5c (Fig. 3a). Crucially, none of these products could be synthesized directly in a one-pot manner by combining two types of amine precursors and the hexaformyl precursor 1. For example, combining 1, *R*-CHDA, and 6b in a 3 : 8 : 2 ratio in CDCl_3_ produced a library of intractable mixtures (Fig. S42, SI). This observation indicated that the successful formation of *R*-5 results from the kinetic stability of *R*-4. That is, the secondary amines (6a–6c) preferentially react with the more accessible formyl units of *R*-4 rather than undergoing kinetically disfavored imine exchange with the *R*-CHDA residues, each bearing two imine connections with the macrocycle framework.

## Conclusions

To summarize, by leveraging the dynamic nature of imine bonds, an unexpected chiral macrocycle with a non-closed structure was serendipitously obtained as the sole observed product by the condensation of a chiral diamine and a hexaformyl precursor. Unlike conventional self-assembled systems where all amino and formyl groups form imine bonds, this macrocycle retains two unreacted amino groups. DFT calculations demonstrated that the gibbs free energy of this structure is significantly lower than that of the predicted tube-shaped structure. Crucially, the macrocycle contains two diamine residues, each connected to the framework *via* only one imine linkage. These residues are consequently susceptible to removal *via* imine exchange or hydrolysis, yielding a non-closed macrocycle with two unreacted formyl groups. This resulting macrocycle can then serve as a diformyl precursor for hierarchically upgraded self-assembly, enabling the construction of more complex architectures comprising multiple types of amine or formyl building blocks, whose construction would otherwise be challenging to access *via* one-pot self-assembly.

## Author contributions

H. L. conceived the project. Z. C., C. G., G. W. and H. L. prepared the manuscript. Z. C. and C. G. synthesized the molecules studied in this work. Z. C. and C. G. performed characterization of the key compounds. Z. C., C. G., G. W. and H. L. analyzed experimental data and draw conclusions. G. W. performed theoretical calculations and analysis. J. L. determined the crystal structure. All authors discussed the results and commented on the manuscript.

## Conflicts of interest

There are no conflicts to declare.

## Supplementary Material

SC-017-D5SC06544E-s001

SC-017-D5SC06544E-s002

## Data Availability

The data supporting this article have been included as part of the supplementary information (SI). Supplementary information is available. See DOI: https://doi.org/10.1039/d5sc06544e. CCDC 2446596: Experimental Crystal Structure, 2025.^52^
