# Supplementary material for: A self-assembled macrocycle with a non-closed structure for hierarchically upgraded self-assembly
Source: Chem Sci. 2025 Nov 11;17(1):381–6. doi: 10.1039/d5sc06544e (PMC12606462; doi:10.1039/d5sc06544e)
Supplement: SC-017-D5SC06544E-s001 [file SC-017-D5SC06544E-s001.pdf]

Supplementary Information (SI) for Chemical Science.
This journal is © The Royal Society of Chemistry 2025

Supporting Information

A self-assembled macrocycle with a non-closed structure for hierarchically upgraded self-assembly

Ze Cao[+], Chenqi Ge[+], Guangcheng Wu[+]*, Hua Tang, Yating Wu, Yueyan Kuang, Yuyang Lu, Jiyong Liu and Hao Li*

1. Materials and General Methods S2
2. Synthetic Procedures S2
3. NMR Spectra and HRMS Spectra S7
4. The Yields of *R*-**4** to *R*-**2** and *R*-**5** S22
5. UV/Vis and Circular Dichroism Spectra S25
6. X-ray Crystallography S25
7. Theoretical Calculations S27
8. Reaction of *R*-**4** with Amines S29
9. References S33

1. Materials and General Methods

All reagents and solvents were purchased from commercial sources and used without further purification. The chloroform solvent was sourced from Sinopharm Chemical Reagent Co., Ltd. (purity of >99%), containing 0.3 – 1.0% ethanol as a stabilizer. It was employed as received, without further distillation or alkali treatment. The deuterated chloroform (CDCl3) was sourced from Energy Chemical (deuterium content 99.8%), containing 0.03% tetramethylsilane (TMS) as an internal reference. Likewise, it was used directly without any purification. The Manipulations were performed under an ambient condition unless otherwise indicated. Nuclear magnetic resonance (NMR) spectra were recorded at ambient temperature using Bruker AVANCE III 500 and Agilent DD2 600 spectrometers, with working frequencies of 500/600 and 125/150 MHz for ^{1}H and ^{13}C, respectively. Chemical shifts are reported in ppm relative to the residual internal non-deuterated solvent signals ($CDCl_3$: δ = 7.26 ppm, CD_2Cl_2: δ = 5.32 ppm, $C_2D_2Cl_4$: δ = 5.98 ppm). High-resolution mass spectra (HRMS) were recorded on an Agilent 6545 Quadrupole TOF MS. X-ray crystallographic data were collected on a Bruker D8 Venture diffractometer. CD spectra were recorded on a Circular Dichroism Spectrometer (J-1500, JASCO).

2. Synthetic Procedures

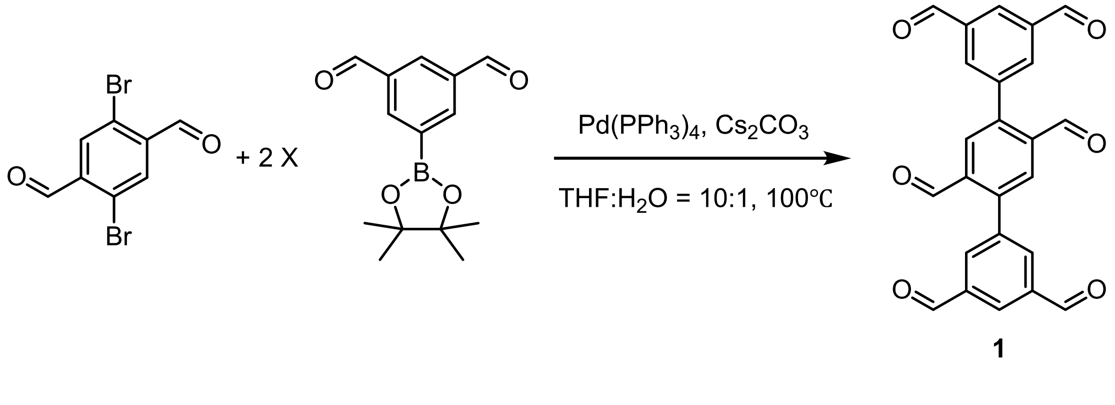

Scheme S1. Synthesis of **1**.

1: 2,5-dibromoterephthalaldehyde (0.292 g, 1.0 mmol), 5-(4,4,5,5-tetramethyl-1,3,2-dioxaborolan-2-yl)isophthalaldehyde (0.624 g, 2.4 mmol), cesium carbonate (1.30 g,

4.0 mmol), tetrakis(triphenylphosphine)palladium (58 mg, 0.050 mmol), tetrahydrofuran (THF) (10 mL) and water (1 mL) were combined in a 35 mL Schlenk tube and stirred at 100 °C under an argon atmosphere. After 16 h, the reaction mixture was cooled to room temperature. The precipitates were collected *via* filtration, which were then washed with tetrahydrofuran, water, acetone and finally dried under vacuum, which afforded the crude product **1**, which was used in self-assembly without further purification due to its poor solubility.

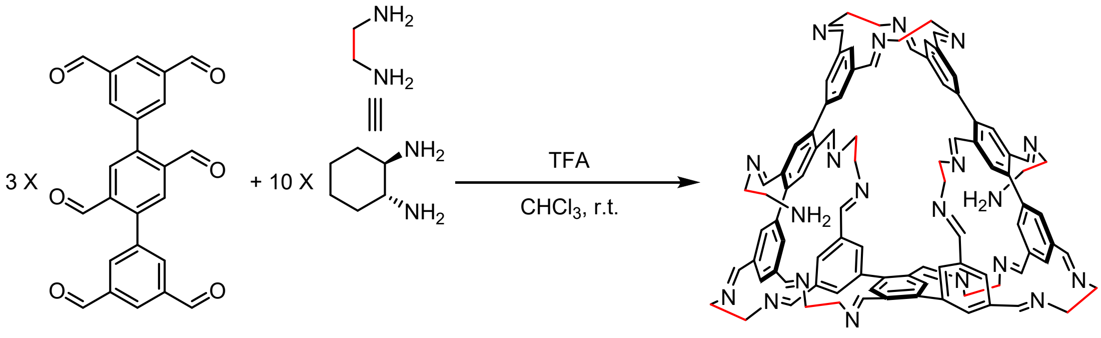

Scheme S2. Synthesis of *R*-**2**.

R-**2**: **1** (0.199 g, 0.50 mmol), (*R*, *R*)-1,2-diaminocyclohexane (*R*-CHDA) (0.228 g, 2.0 mmol) and a catalytic amount of trifluoroacetic acid (TFA) (0.05 mL) were combined in 500 mL $CHCl_3$ and stirred at room temperature for 24 h. **1** was gradually dissolved, in spite of a little insoluble material, which was filtered off. The filtrate was then removed under vacuum. The solid-state material was re-dissolved in a small amount of $CHCl_3$, and MeOH was then added, yielding *R*-**2** as a white powder (0.094 g, 31 % in two steps relative to 2,5-dibromoterephthalaldehyde). *R*-**2** was characterized by recording its ^{1}H NMR spectrum (Fig. S1), ^{13}C NMR spectrum (Fig. S2), ^{1}H-^{1}H COSY spectrum (Fig. S3), ^{1}H-^{1}H ROESY spectrum (Fig. S4 and S5), DOSY spectrum (Fig. S6) and mass spectrum (Fig. S7). **^{1}H NMR** (600 MHz, CD_2Cl_2) δ (ppm): 8.53 (s, 2H), 8.40 (s, 2H), 8.39 (s, 2H), 8.35 (s, 2H), 8.21 (s, 2H), 8.06 (s, 2H), 8.04 (s, 2H), 8.02 (s, 2H), 7.99 (s, 2H), 7.95 (s, 2H), 7.90 (s, 2H), 7.89 (s, 2H), 7.81 (s, 2H), 7.76 (s, 2H), 7.58 (s, 2H), 7.46 (s, 2H), 7.35 (s, 2H), 7.33 (s, 2H), 7.14 (s, 2H), 6.80 (s, 2H), 6.51 (s, 2H), 3.62 – 3.56 (q, 2H), 3.56 – 3.50 (m, 2H), 3.37 – 3.27 (m, 6H), 3.26 – 3.20 (m, 2H), 3.11 – 3.02 (m, 4H), 2.74 – 2.68 (m, 2H), 2.62 – 2.55 (m, 2H), 2.44 (d, 2H), 2.25 – 1.01 (m, 82H). **^{13}C NMR** (150 MHz, CD_2Cl_2) δ (ppm): 162.7, 162.0, 161.8, 160.9, 160.7,

160.1, 159.9, 159.8, 159.1, 141.9, 140.3, 140.3, 139.7, 139.1, 138.8, 137.7, 137.6, 137.5, 136.8, 136.7, 136.3, 136.3, 135.9, 135.5, 133.6, 132.9, 132.0, 131.3, 130.7, 129.8, 128.7, 128.6, 128.2, 127.5, 127.4, 127.0, 79.6, 78.6, 78.1, 76.4, 75.1, 75.0, 74.6, 74.1, 71.8, 55.2, 35.6, 34.0, 33.4, 33.3, 32.8, 32.4, 32.2, 32.2, 32.0, 30.1, 26.9, 25.8, 25.2, 25.1, 25.0, 25.0, 24.9, 24.9, 24.9, 24.7. As *R*-**2** exhibits C_2 symmetry, the number of ^{13}C signals was expected to be 66, and the number of ^{13}C signals observed was 66, which is consistent with expectations. **ESI-HRMS**: *m/z* calculated for $C_{132}H_{148}N_{20}^{2+}$ ([*R*-**2**+2H]$^{2+}$): 1006.6093; found: 1006.6086. $C_{132}H_{147}N_{20}Na^{2+}$ ([*R*-**2**+H+Na]$^{2+}$): 1017.6002; found: 1017.5999. $C_{132}H_{147}N_{20}^{+}$ ([*R*-**2**+H]$^{+}$): 2012.2112; found: 2012.2103. $C_{132}H_{148}N_{20}Na^{+}$ ([*R*-**2**+Na]$^{+}$): 2034.1932; found: 2034.1904.

S-**2**: **1** (0.199 g, 0.50 mmol), (*S*, *S*)-1,2-diaminocyclohexane (*S*-CHDA) (0.228 g, 2.0 mmol) and a catalytic amount of trifluoroacetic acid (TFA) (0.05 mL) were combined in 500 mL $CHCl_3$ and stirred at room temperature for 24 h. **1** was gradually dissolved, in spite of a little insoluble material, which was filtered off. The filtrate was then removed under vacuum. The solid-state material was re-dissolved in a small amount of $CHCl_3$, and MeOH was then added, yielding *S*-**2** as a white powder (0.103 g, 34 % in two steps relative to 2,5-dibromoterephthalaldehyde).

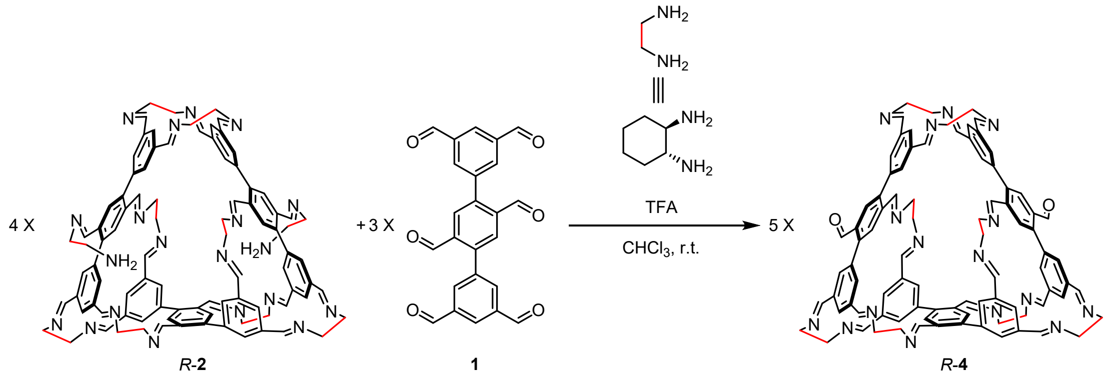

Scheme S3. Synthesis of *R*-**4**.

R-**4**: *R*-**2** (0.080 g, 0.04 mmol), **1** (0.064 g, 0.16 mmol) and a catalytic amount of trifluoroacetic acid (TFA) (1 μL) were combined in 10 mL $CHCl_3$ and stirred at room temperature for 48 h. **1** was gradually dissolved, in spite of a little insoluble material, which was filtered off. The filtrate was then removed under vacuum. The solid-state material was re-dissolved in a small amount of $CHCl_3$, and MeOH was then added, yielding *R*-**4** as a white powder (0.084 g, 92 % relative to *R*-**2**). *R*-**4** was characterized

by recording its ^{1}H NMR spectrum (Fig. S15), ^{13}C NMR spectrum (Fig. S16), ^{1}H-^{1}H COSY spectrum (Fig. S17), ^{1}H-^{1}H ROESY spectrum (Fig. S18 and S19), DOSY spectrum (Fig. S20) and mass spectrum (Fig. S21). **^{1}H NMR** (600 MHz, $CDCl_3$) δ (ppm): 10.00 (s, 2H), 8.55 (s, 2H), 8.44 (s, 2H), 8.41 (s, 2H), 8.36 (s, 2H), 8.05 (s, 2H), 8.04 (s, 2H), 8.03 (s, 2H), 7.99 (s, 4H), 7.93 (s, 2H), 7.91 (s, 2H), 7.80 (s, 4H), 7.74 (s, 2H), 7.40 (s, 2H), 7.37 (s, 2H), 7.34 (s, 2H), 7.30 (s, 2H), 6.89 (s, 2H), 6.60 (s, 2H), 3.63 – 3.57 (q, 2H), 3.57 – 3.50 (m, 2H), 3.39 – 3.30 (m, 6H), 3.24 – 3.17 (m, 4H), 3.10 – 3.04 (m, 2H), 2.44 (d, 2H), 2.18 – 1.16 (m, 62H). **^{13}C NMR** (150 MHz, $CDCl_3$) δ (ppm): 190.2, 160.9, 160.4, 160.3, 159.8, 159.7, 159.1, 158.6, 158.4, 143.4, 139.1, 138.9, 138.3, 137.9, 136.8, 136.7, 136.3, 136.1, 135.9, 135.6, 135.0, 134.4, 134.3, 133.2, 132.7, 131.8, 131.1, 130.8, 129.5, 128.9, 128.0, 127.3, 127.0, 126.5, 126.4, 126.0, 77.2, 76.9, 74.9, 73.8, 73.8, 73.7, 72.1, 70.8, 31.9, 31.4, 31.4, 31.2, 30.8, 30.7, 30.6, 30.6, 28.7, 25.4, 23.7, 23.7, 23.7, 23.4, 23.4, 23.3. As *R*-**4** exhibits C_2 symmetry, the number of ^{13}C signals was expected to be 60, and the number of ^{13}C signals observed was 60, which is consistent with expectations. **ESI-HRMS**: *m/z* calculated for $C_{120}H_{124}N_{16}O_2^{2+}$ ([*R*-**4**+2H]$^{2+}$): 910.5041; found: 910.5041. $C_{120}H_{123}N_{16}O_2^{+}$ ([*R*-**4**+H]$^{+}$): 1820.0009; found: 1820.0006.

S-**4**: *S*-**2** (0.080 g, 0.04 mmol), **1** (0.064 g, 0.16 mmol) and a catalytic amount of trifluoroacetic acid (TFA) (1 μL) were combined in 10 mL $CHCl_3$ and stirred at room temperature for 48 h. **1** was gradually dissolved, in spite of a little insoluble material, which was filtered off. The filtrate was then removed under vacuum. The solid-state material was re-dissolved in a small amount of $CHCl_3$, and MeOH was then added, yielding *S*-**4** as a white powder (0.080 g, 87 % relative to *S*-**2**).

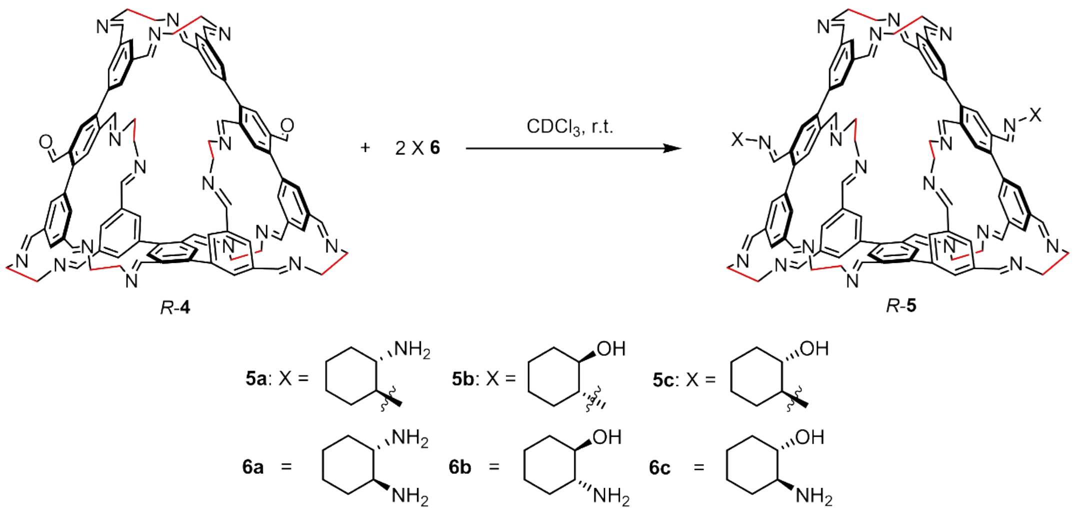

Scheme S4 Synthesis of *R*-**5a,** *R*-**5b** and *R*-**5c**.

R-**5a**: *R*-**4** (4.5 mg, 0.0025 mmol) and **6a** (2.85 mg, 0.025 mmol) were combined in 1 mL $CDCl_3$ and stirred at room temperature for 24 h to form *R*-**5a** (72% relative to *R*-**4**, determined using 1,2-dichloroethane as an internal standard in $CDCl_3$, Figure S31). *R*-**5a** was characterized by recording its ^{1}H NMR spectrum (Fig. S23), ^{13}C NMR spectrum (Fig. S24), ^{1}H-^{1}H COSY spectrum (Fig. S25), ^{1}H-^{1}H ROESY spectrum (Fig. S26 and S27), DOSY spectrum (Fig. S28) and mass spectrum (Fig. S29). **^{1}H NMR** (600 MHz, $CDCl_3$) δ (ppm): 8.56 (s, 2H), 8.44 (s, 2H), 8.38 (s, 2H), 8.36 (s, 2H), 8.27 (s, 2H), 8.07 (s, 2H), 8.03 (s, 2H), 8.02 (s, 2H), 7.96 (s, 2H), 7.95 (s, 2H), 7.89 (s, 2H), 7.87 (s, 2H), 7.82 (s, 2H), 7.80 (s, 2H), 7.67 (s, 2H), 7.51 (s, 2H), 7.37 (s, 2H), 7.34 (s, 2H), 7.23 (s, 2H), 6.84 (s, 2H), 6.52 (s, 2H), 3.63 – 3.58 (m, 2H), 3.56 – 3.50 (m, 2H), 3.36 – 3.27 (m, 6H), 3.25 – 3.19 (m, 4H), 3.06 – 2.99 (m, 2H), 2.87 – 2.81 (m, 2H), 2.68 – 2.62 (m, 2H), 2.52 – 2.46 (d, 2H), 2.18 – 1.16 (m, 82H). **^{13}C NMR** (150 MHz, $CDCl_3$) δ (ppm): 162.1, 161.9, 161.5, 161.4, 160.6, 160.5, 160.0, 159.6, 159.2, 141.4, 140.1, 139.9, 139.3, 138.5, 138.4, 137.1, 136.8, 136.7, 136.5, 136.2, 135.7, 135.6, 135.3, 135.3, 133.9, 132.8, 131.7, 131.3, 130.6, 129.9, 128.2, 128.2, 128.1, 127.4, 127.0, 126.6, 78.3, 77.5, 75.9, 74.8, 74.7, 74.6, 73.0, 72.0, 54.8, 33.6, 33.0, 32.9, 32.4, 32.3, 32.3, 31.8, 31.7, 31.6, 29.7, 26.4, 25.1, 24.8, 24.7, 24.7, 24.5, 24.4, 24.3. As *R*-**5a** exhibits C_2 symmetry, the number of ^{13}C signals was expected to be 66, and the number of ^{13}C signals observed was 63 (after excluding the ^{13}C signals from *S*-CHDA), which is which is three fewer than expected. This discrepancy is likely attributed to the necessity of

performing in-situ ^1H NMR at a relatively low concentration with an excess of *S*-CHDA to obtain *R*-**5a**. Consequently, some signals of *R*-**5a** might be overlapped by those of the unreacted *S*-CHDA. Although this issue could not be resolved experimentally, the structural integrity of *R*-**5a** is confidently confirmed by the well-resolved signals corresponding to the Schiff base carbons (159 - 163 ppm, 9 peaks) and those in the aromatic region (126 - 142 ppm, 27 peaks). **ESI-HRMS**: *m/z* calculated for $C_{132}H_{148}N_{20}^{2+}$ ([*R*-**5a**+2H]$^{2+}$): 1006.6093; found: 1006.6102. $C_{132}H_{147}N_{20}^{+}$ ([*R*-**5a**+H]$^{+}$): 2012.2112; found: 2012.2113.

R-**5b** (85% relative to *R*-**4**, determined using 1,2-dichloroethane as an internal standard in $CDCl_3$, Figure S32) and *R*-**5c** (79% relative to *R*-**4**, determined using 1,2-dichloroethane as an internal standard in $CDCl_3$, Figure S33) was synthesized in a similar procedure, using **6b** or **6c** as the precursor, respectively.

3. NMR Spectra and HRMS Spectra

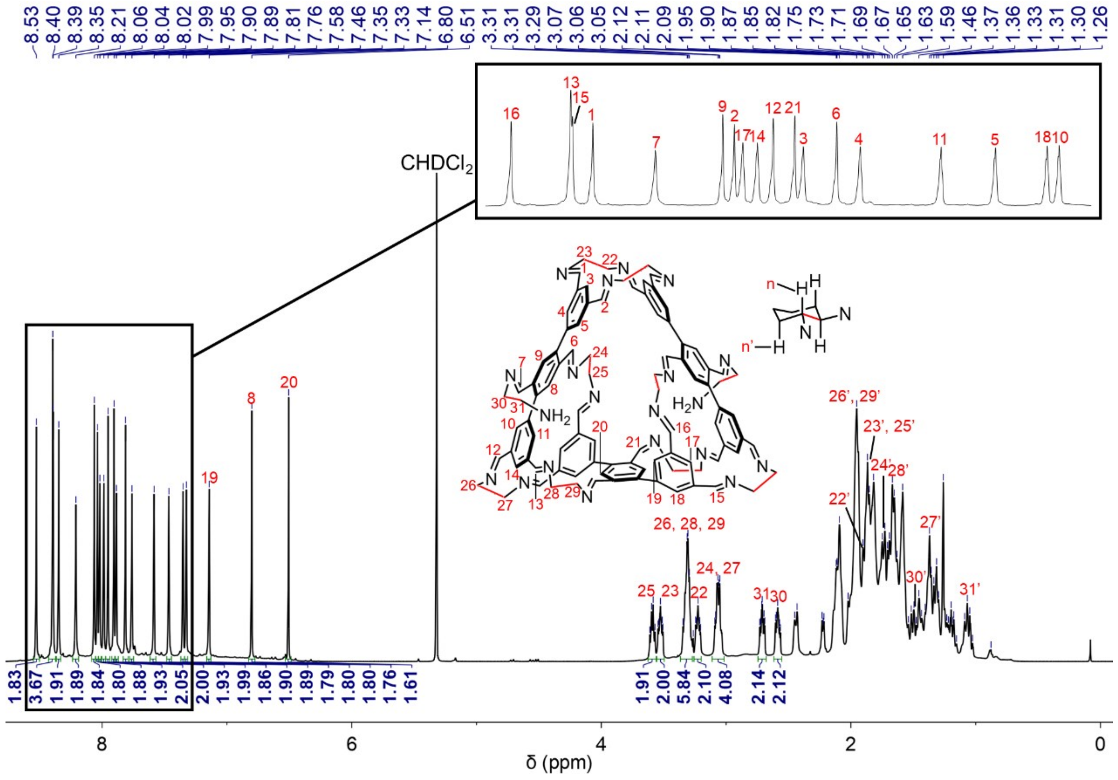

Fig. S1 Partial ^1H NMR spectrum (600 MHz, CD_2Cl_2, 298 K) of *R*-**2**.

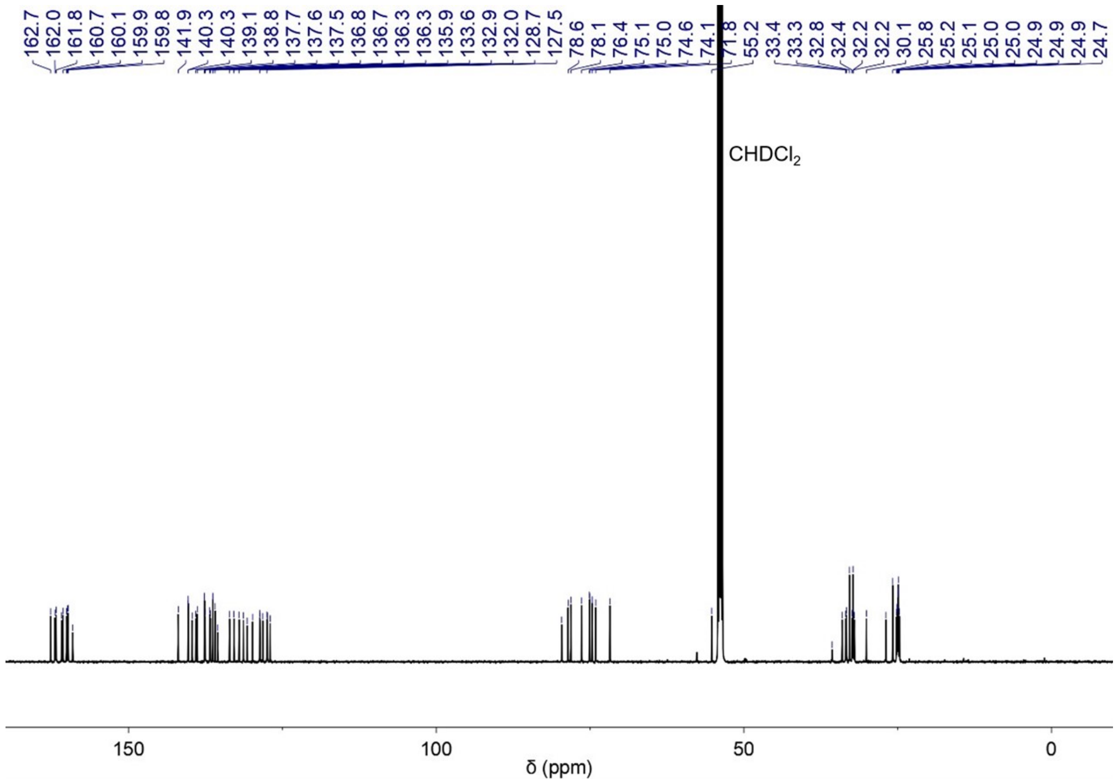

Fig. S2 Partial ^{13}C NMR spectrum (150 MHz, CD_2Cl_2, 298 K) of *R*-**2**.

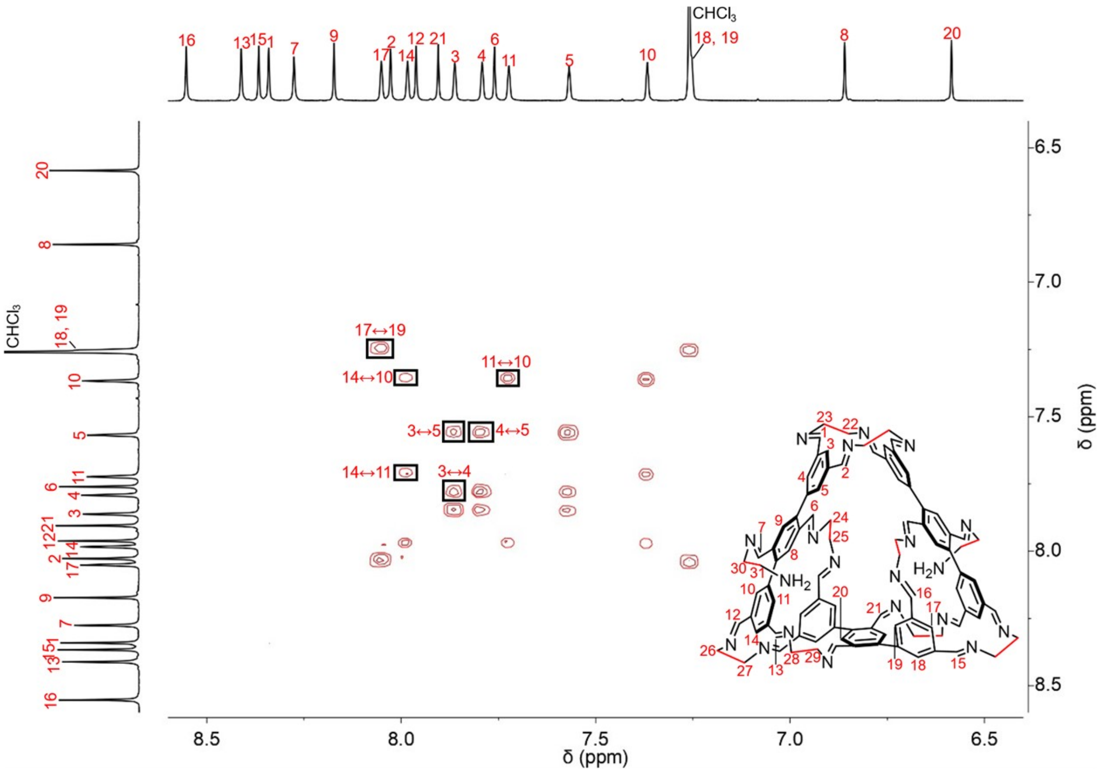

Fig. S3 Partial ^1H-^1H COSY NMR spectrum (600 MHz, $CDCl_3$, 298 K) of *R*-**2**.

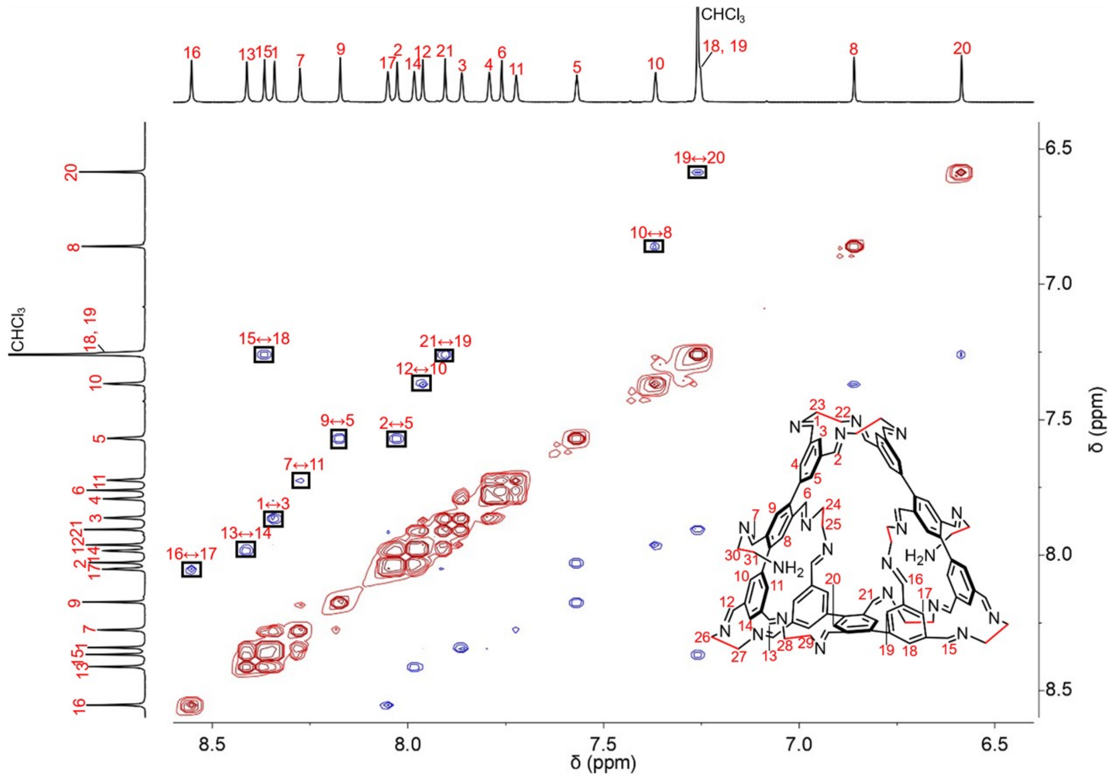

Fig. S4 Partial (Aromatic-aromatic region) ^{1}H-^{1}H ROESY NMR spectrum (600 MHz, $CDCl_3$, 298 K) of *R*-**2**.

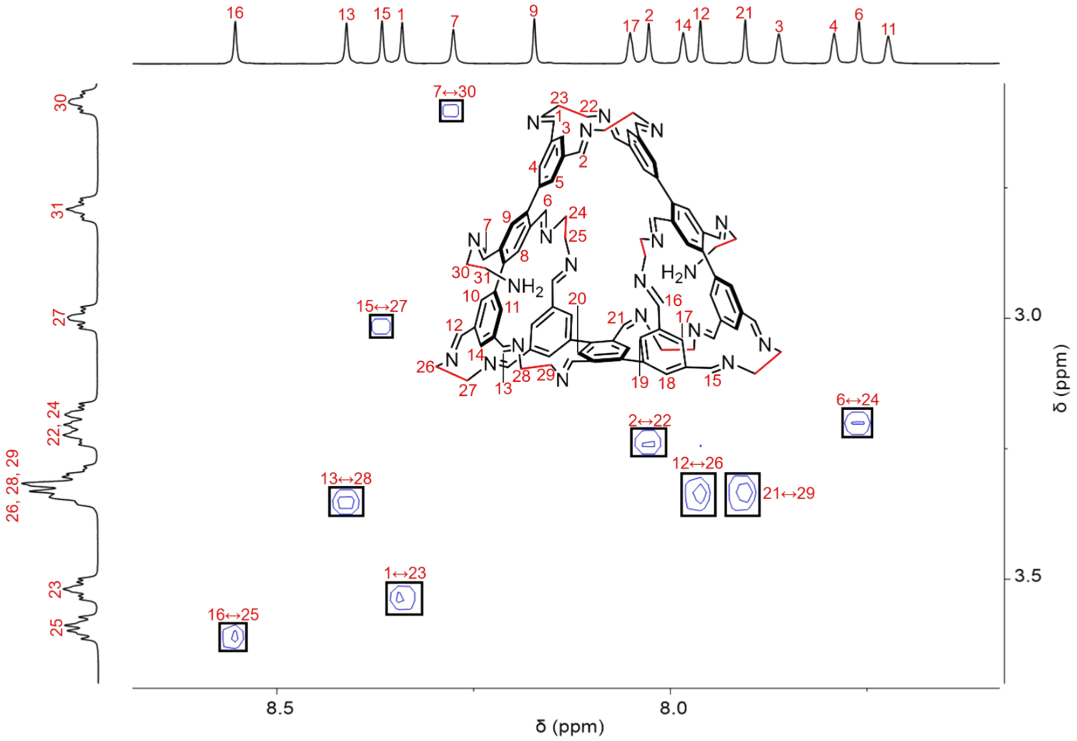

Fig. S5 Partial (Aromatic-aliphatic region) ^{1}H-^{1}H ROESY NMR spectrum (600 MHz, $CDCl_3$, 298 K) of *R*-**2**.

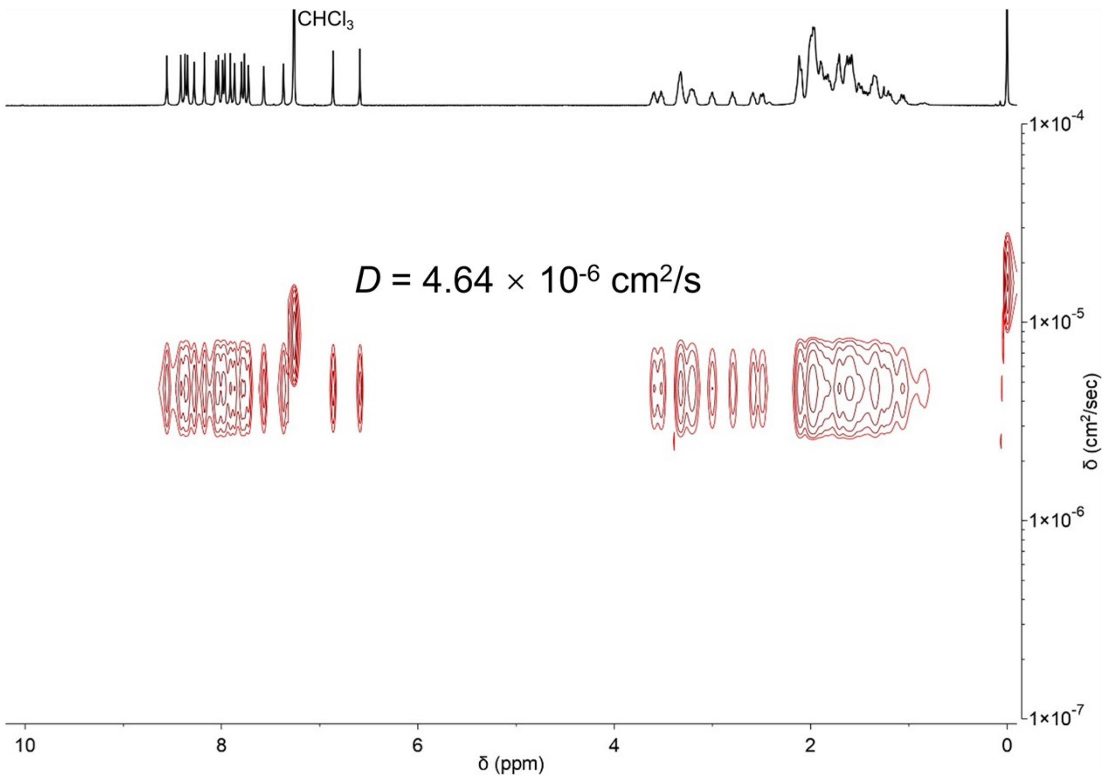

Fig. S6 DOSY spectrum (500 MHz, $CDCl_3$, 298 K) of *R*-**2**. The diffusion coefficient (*D*) was determined to be 4.64×10^{-6} cm^2/s.

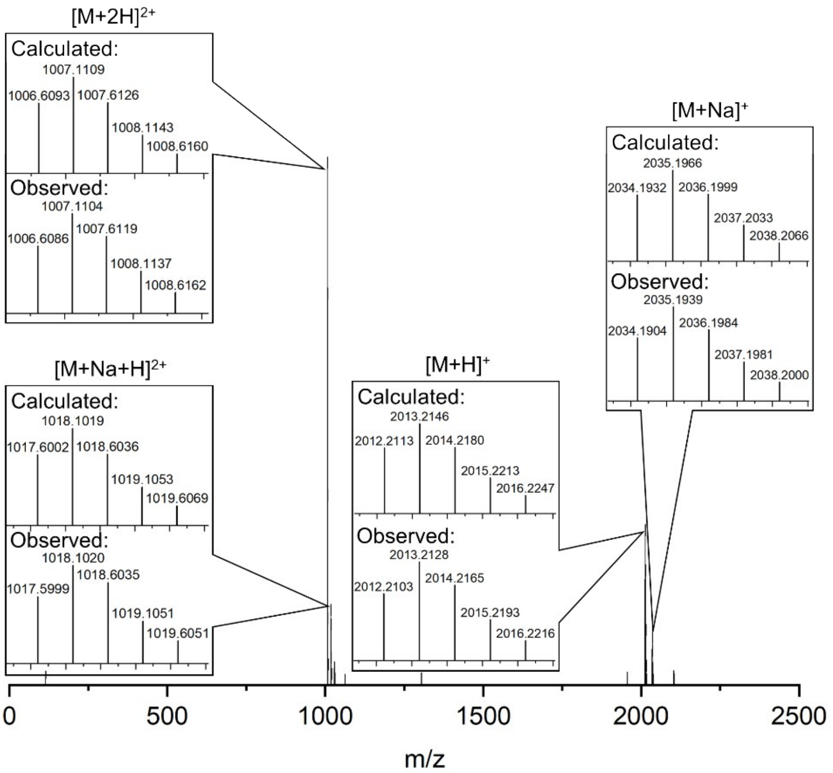

Fig. S7 ESI-HRMS of *R*-**2**.

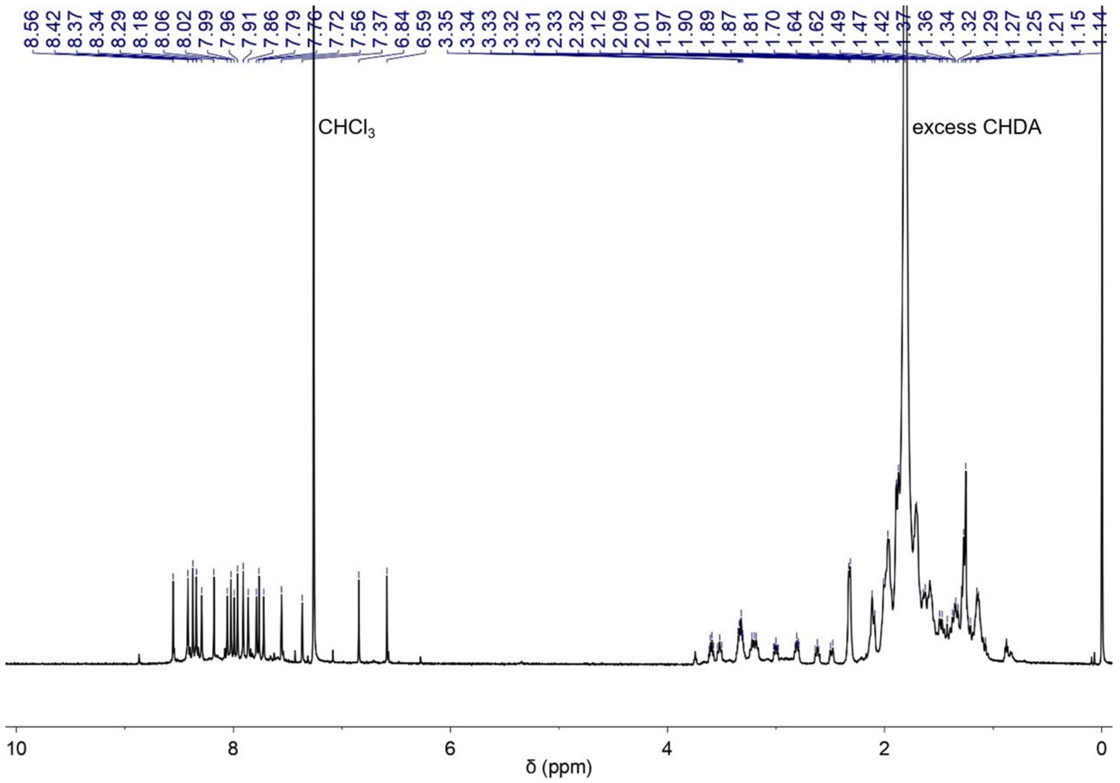

Fig. S8 Partial ^1H NMR spectrum (600 MHz, $CDCl_3$, 298 K) of *R*-**2** in situ.

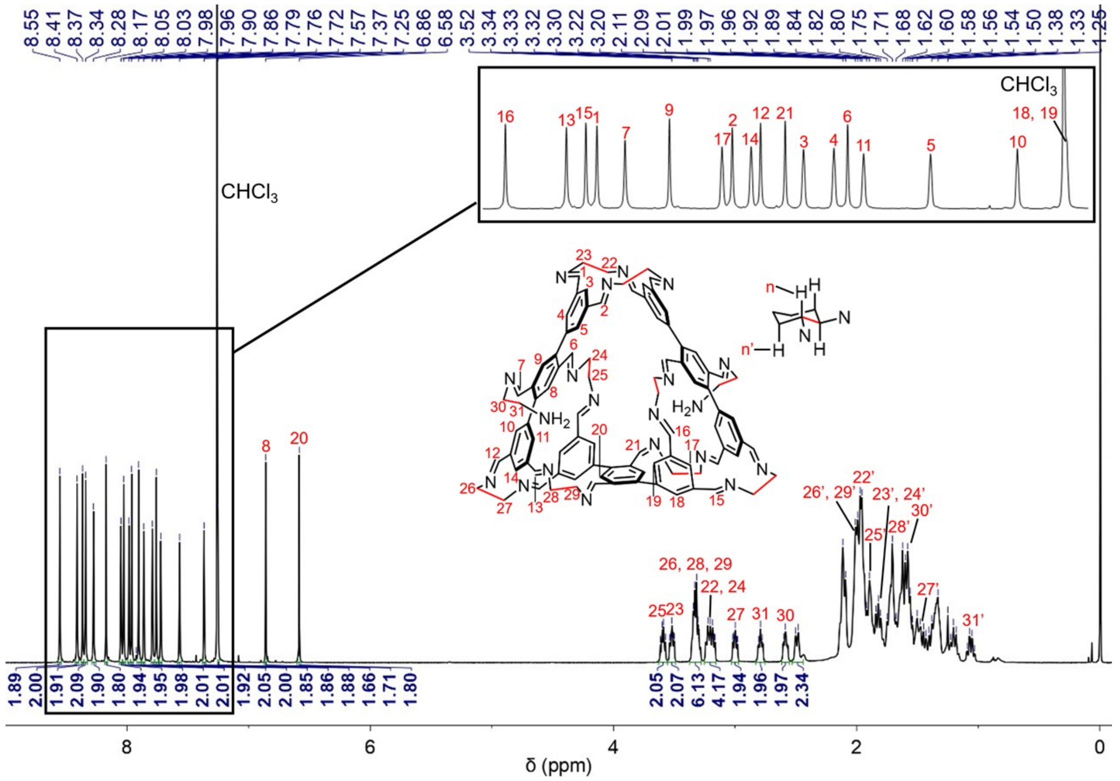

Fig. S9 Partial ^1H NMR spectrum (600 MHz, $CDCl_3$, 298 K) of *R*-**2**.

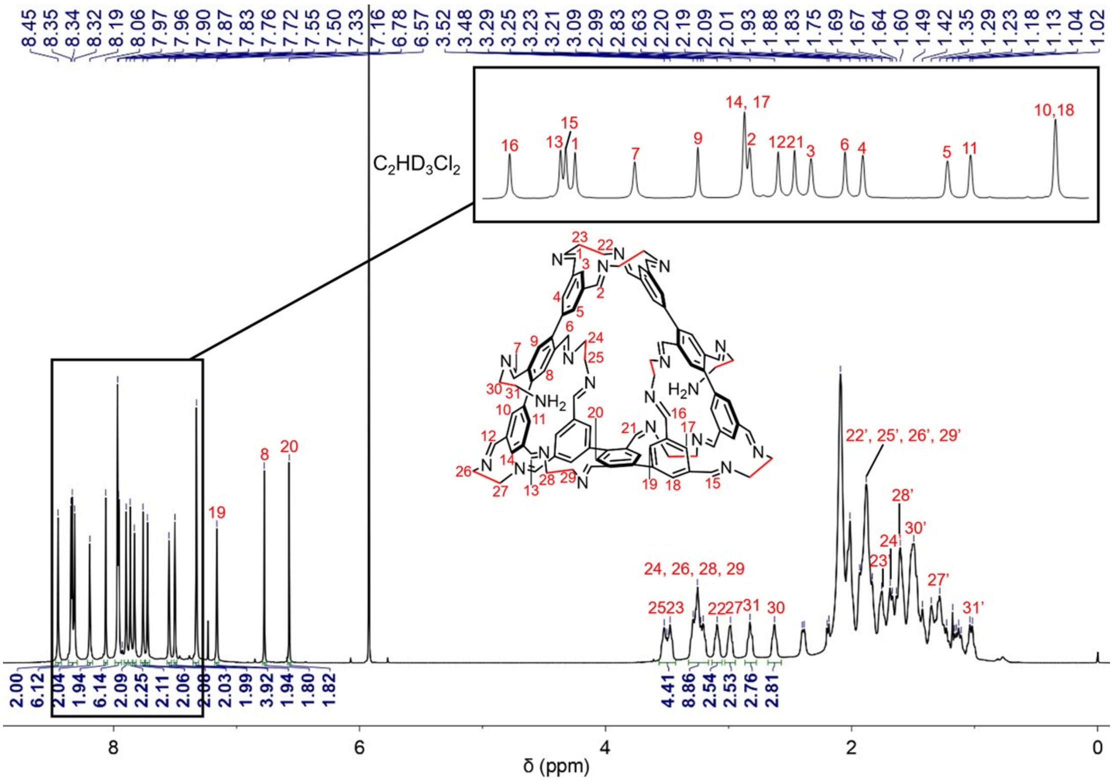

Fig. S10 Partial ^{1}H NMR spectrum (600 MHz, $C_2D_2Cl_4$, 298 K) of *R*-**2**.

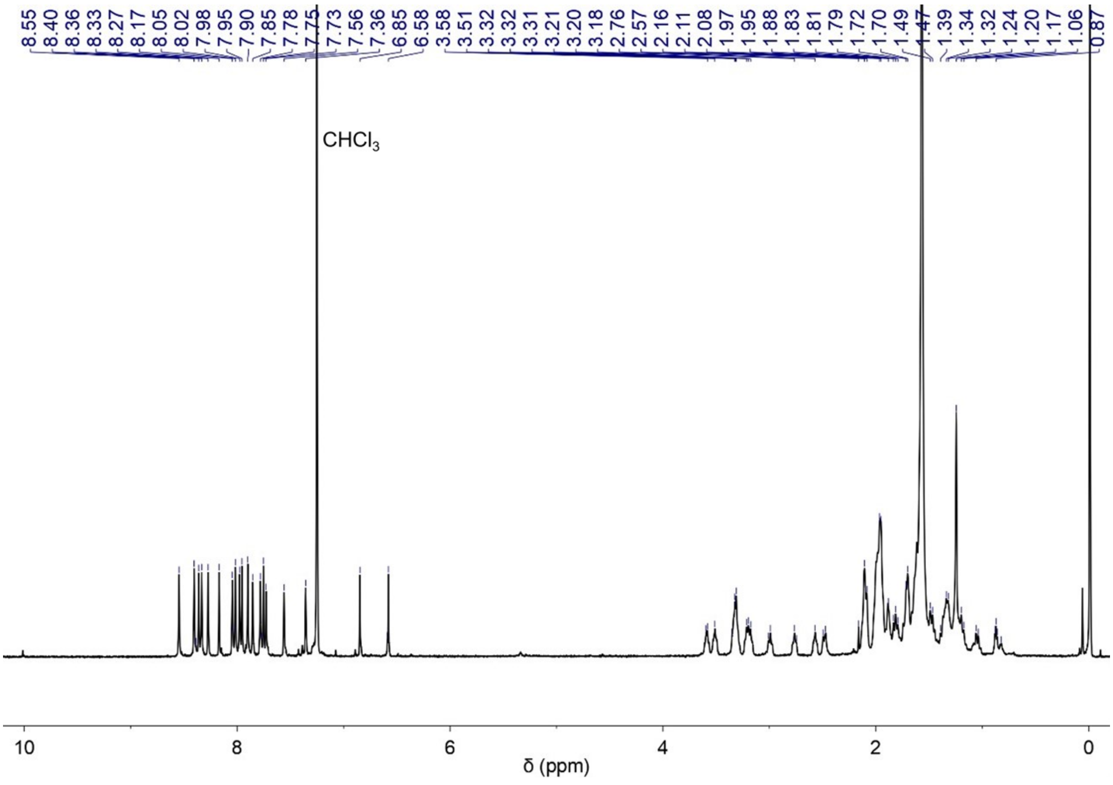

Fig. S11 Partial ^{1}H NMR spectrum (600 MHz, $CDCl_3$, 298 K) of *R*-**2** after three months of storage in $CDCl_3$ solvent.

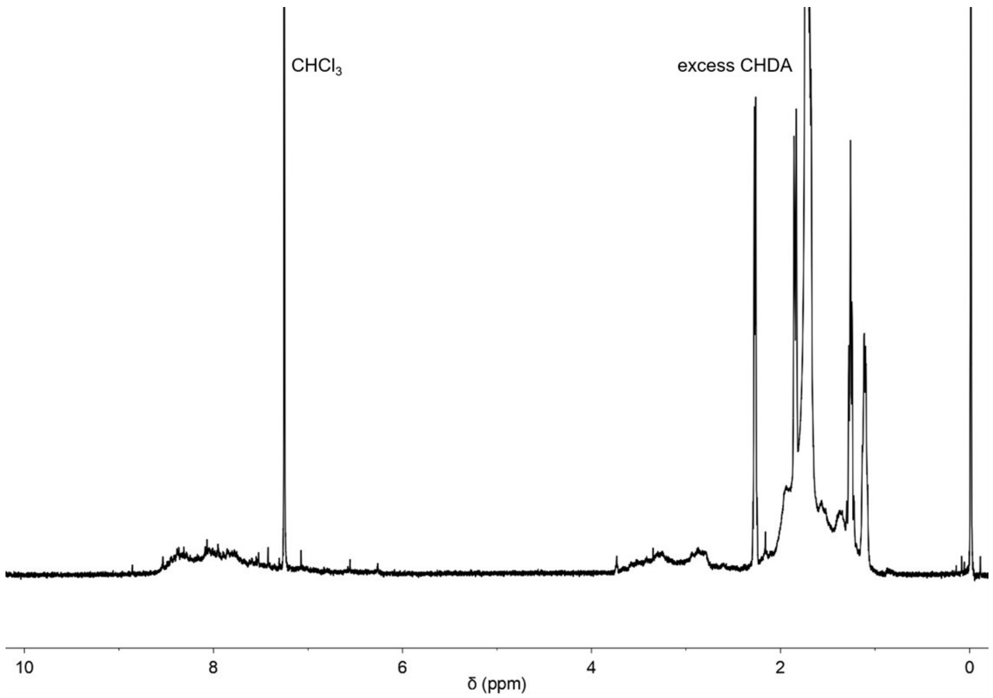

Fig. S12 Partial ^{1}H NMR spectrum (600 MHz, $CDCl_3$, 298 K) of reaction solution of **1** and *rac*-*trans*-CHDA.

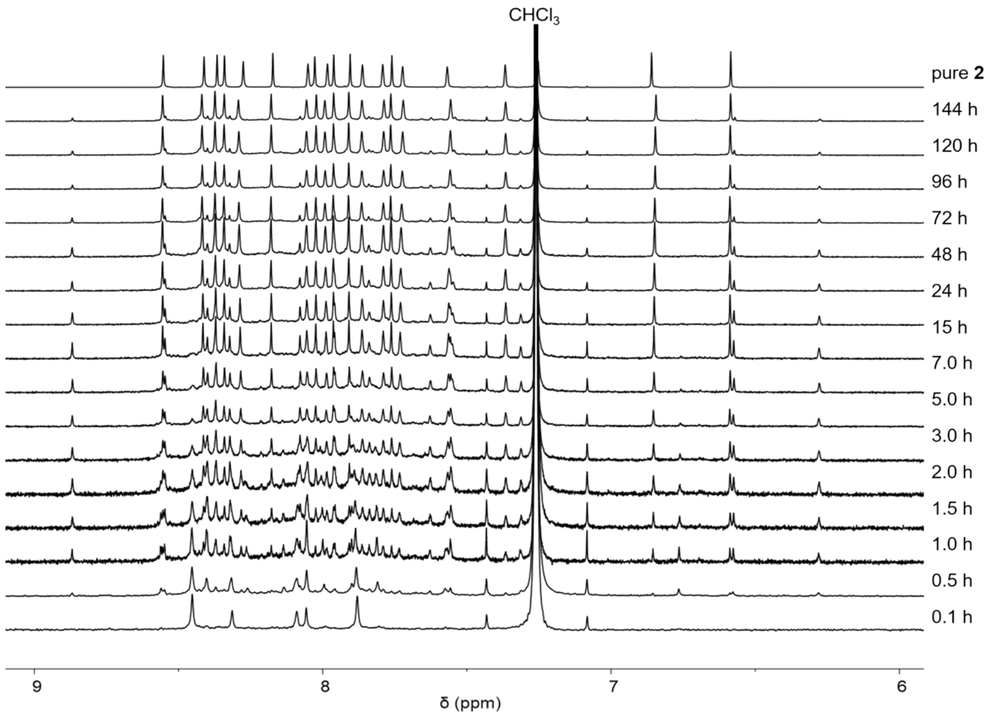

Fig. S13 Partial ^{1}H NMR spectra (600 MHz, $CDCl_3$, 298 K) of combining *R*-CHDA and **1**, which were recorded after certain amount of time. The top spectrum corresponds to pure *R*-**2** isolated *via* precipitation as described in Scheme S2.

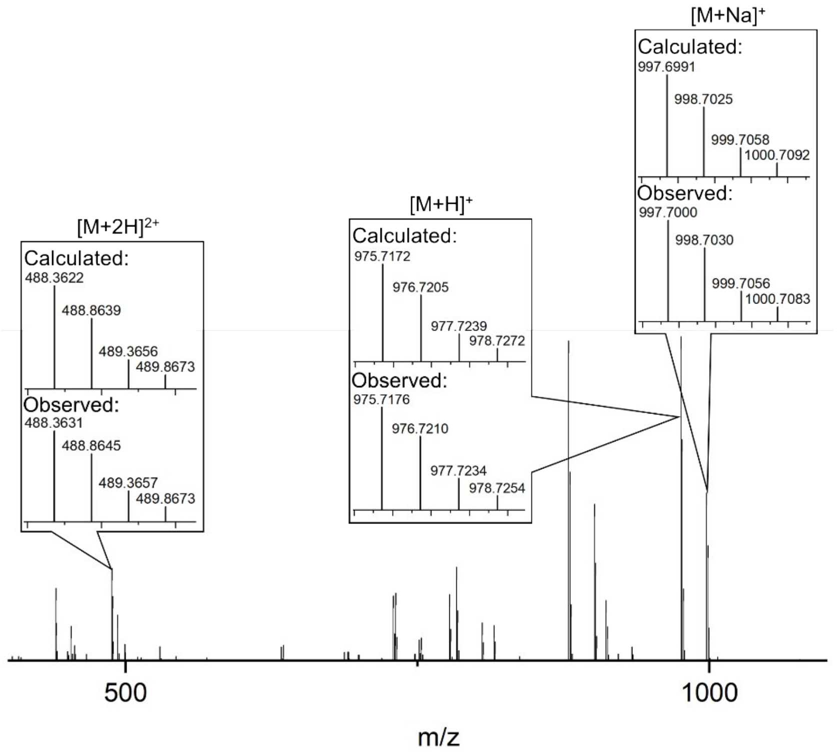

Fig. S14 ESI-HRMS of the early stage self-assembly solution of *R*-**2**. *m/z* calculated for $C_{60}H_{88}N_{12}^{2+}$ ([**M**+2H]$^{2+}$): 488.3622; found: 488.3631; *m/z* calculated for $C_{60}H_{87}N_{12}^{+}$ ([**M**+H]$^{+}$): 975.7171; found: 975.7176. *m/z* calculated for $C_{60}H_{8}N_{12}Na^{+}$ ([**M**+Na]$^{+}$): 997.6991; found: 997.7000.

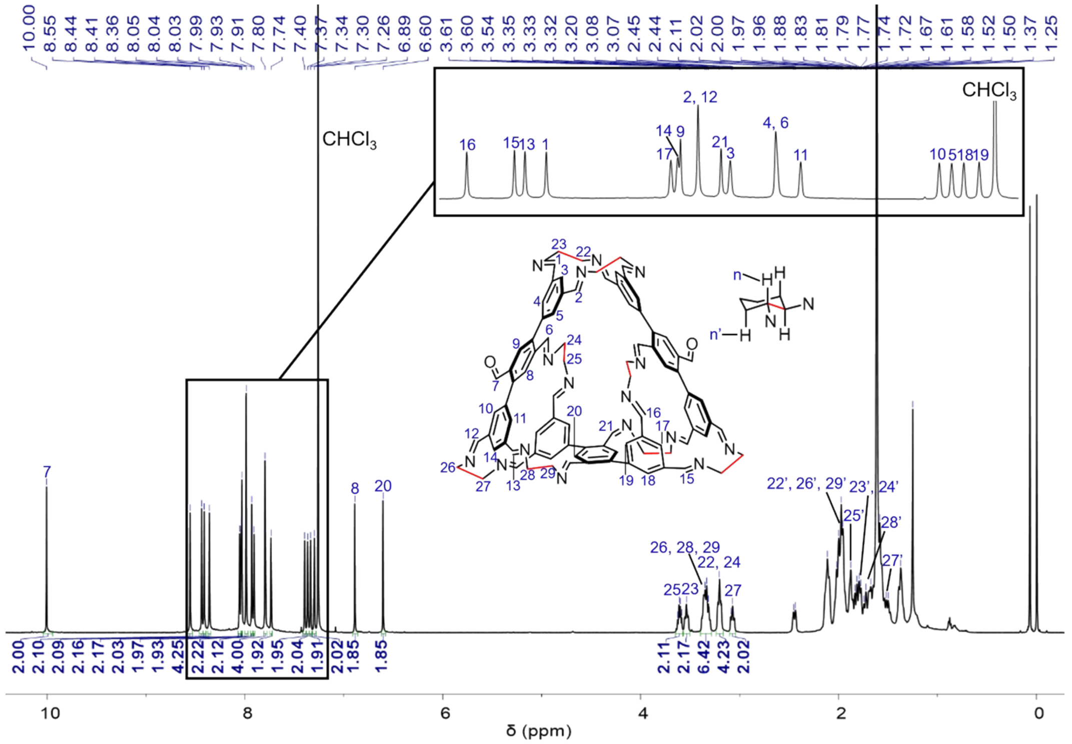

Fig. S15 Partial ^{1}H NMR spectrum (600 MHz, $CDCl_3$, 298 K) of *R*-**4**.

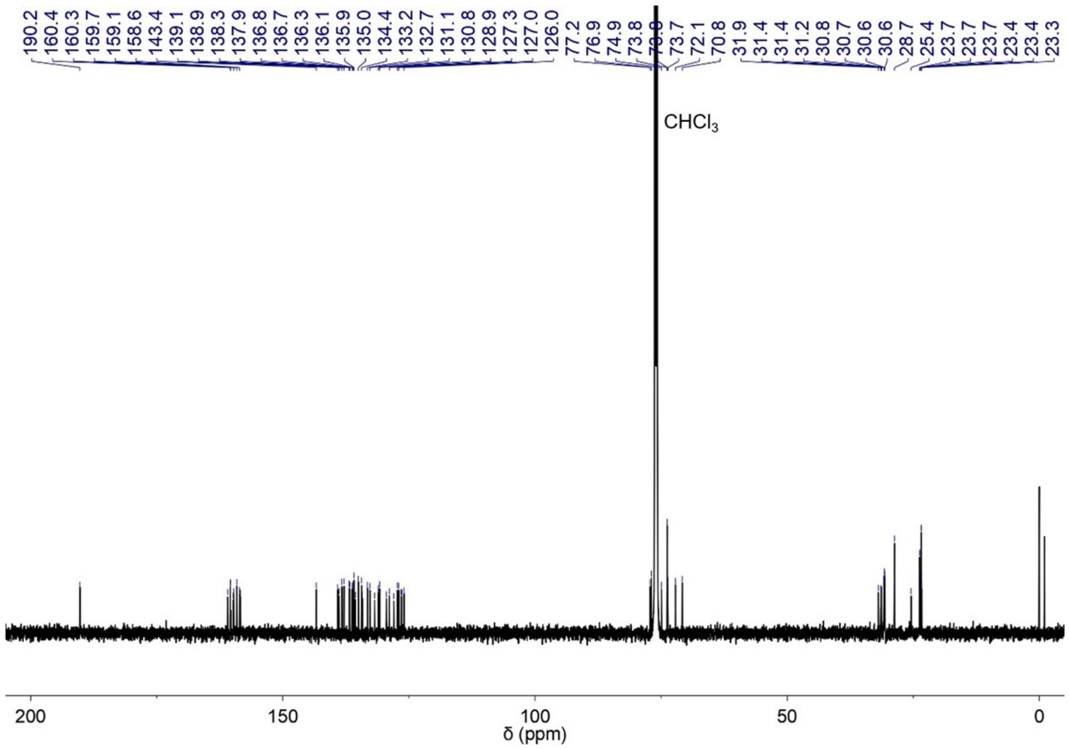

Fig. S16 Partial ^{13}C NMR spectrum (150 MHz, $CDCl_3$, 298 K) of *R*-**4**.

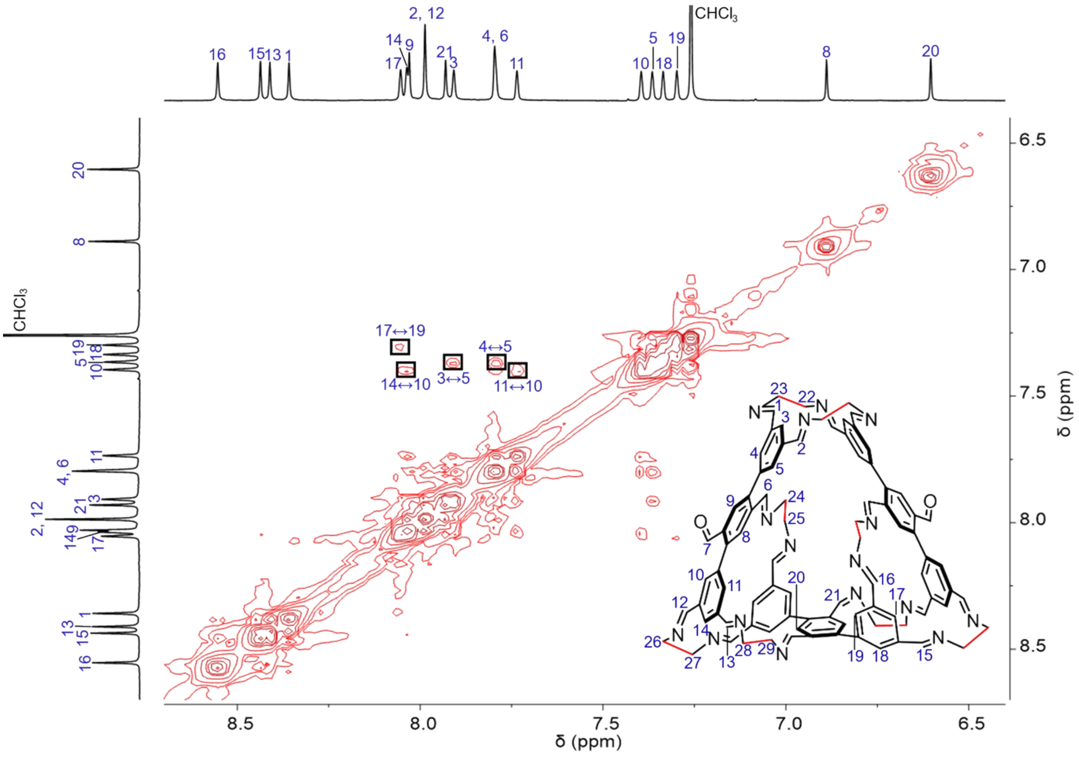

Fig. S17 Partial ^{1}H-^{1}H COSY NMR spectrum (600 MHz, $CDCl_3$, 298 K) of *R*-**4**.

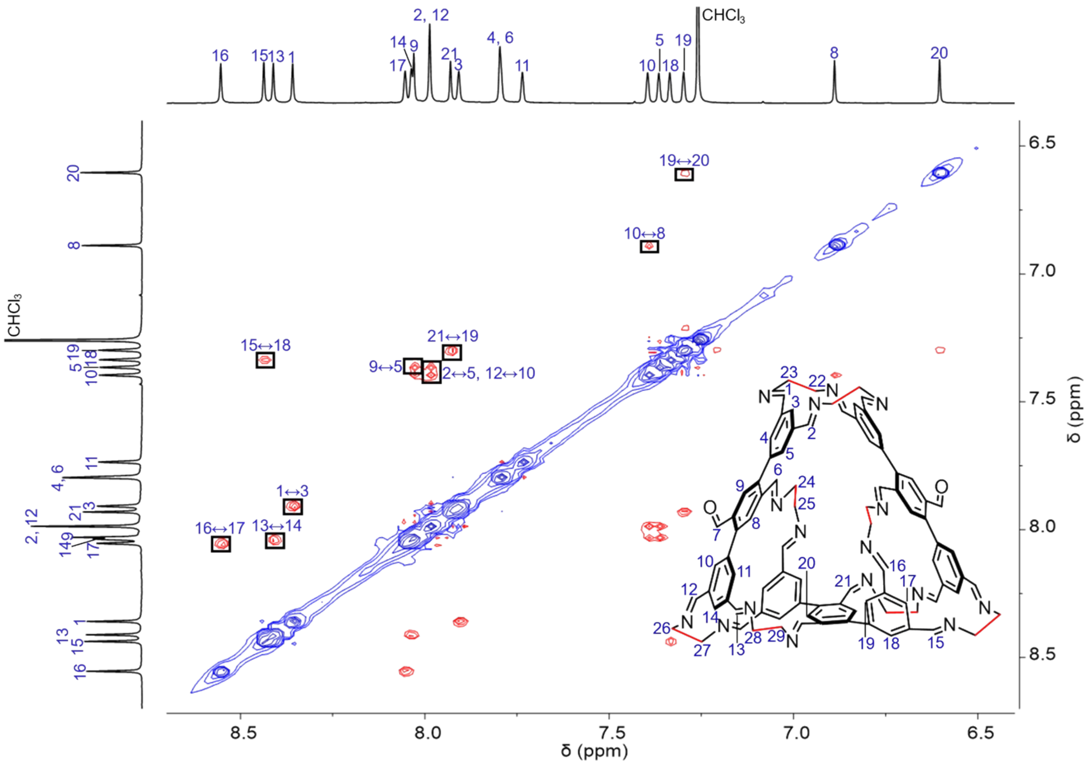

Fig. S18 Partial (Aromatic-aromatic region) ^{1}H-^{1}H ROESY NMR spectrum (600 MHz, $CDCl_3$, 298 K) of *R*-**4**.

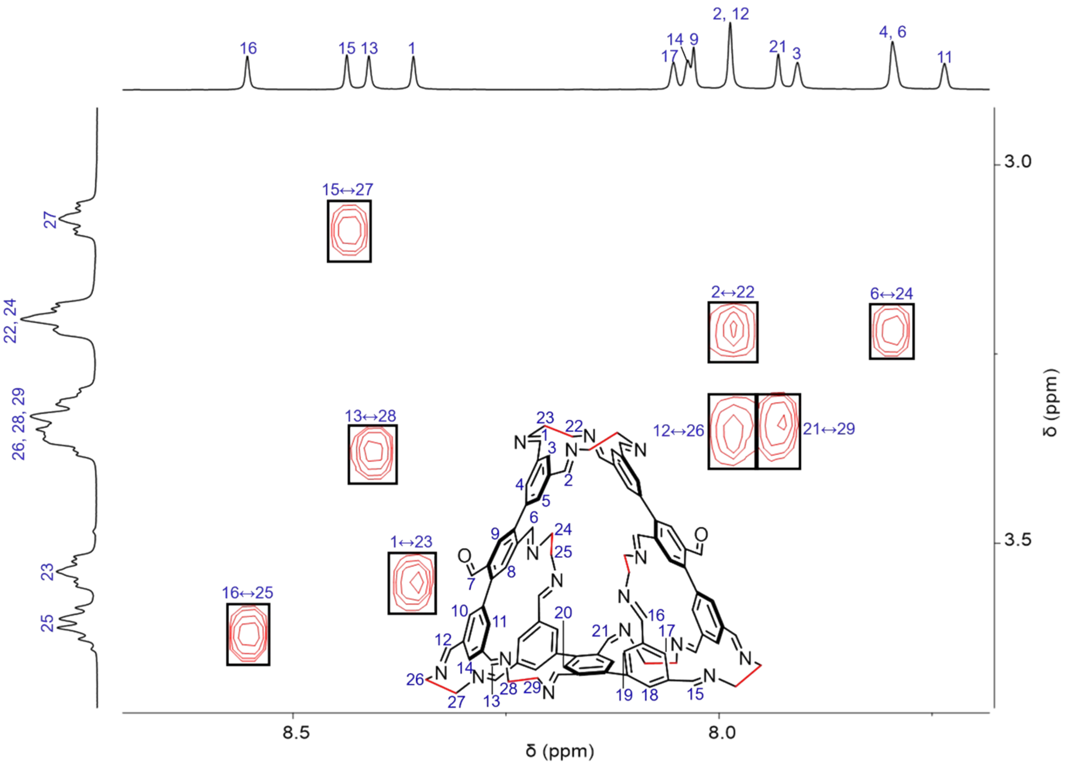

Fig. S19 Partial (Aromatic-aliphatic region) ^{1}H-^{1}H ROESY NMR spectrum (600 MHz, $CDCl_3$, 298 K) of *R*-**4**.

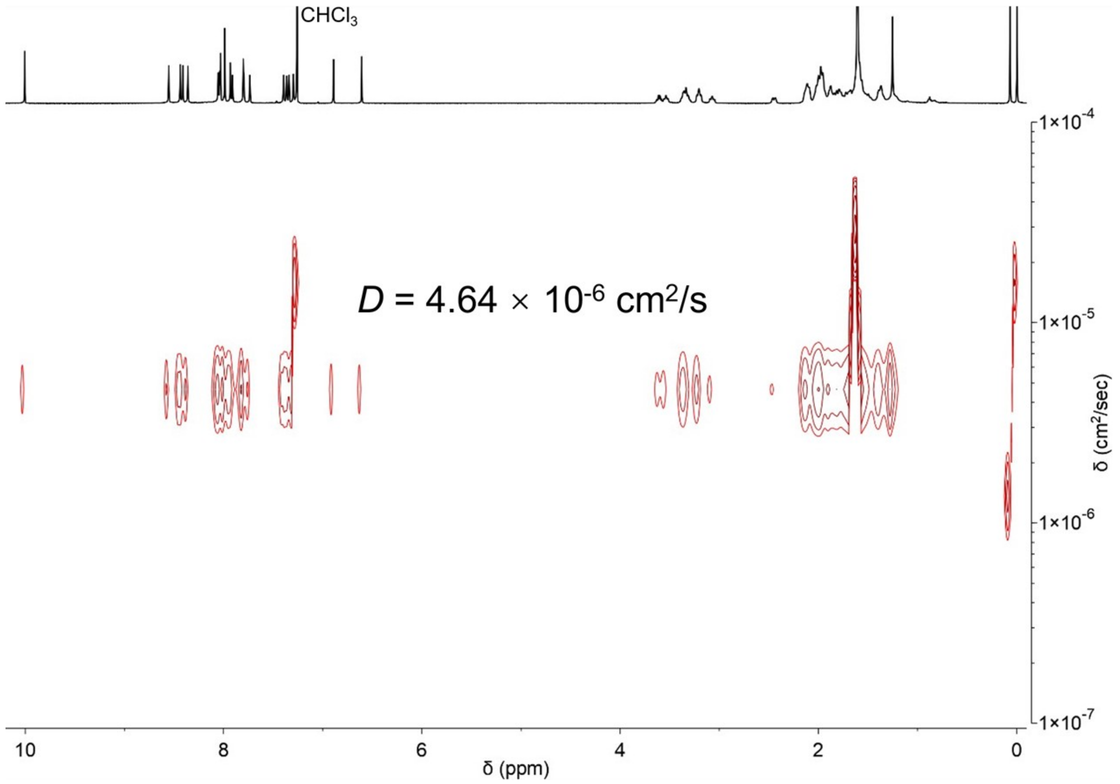

Fig. S20 DOSY spectrum (500 MHz, $CDCl_3$, 298 K) of *R*-**4**. The diffusion coefficient (*D*) was determined to be 4.64×10^{-6} cm^2/s.

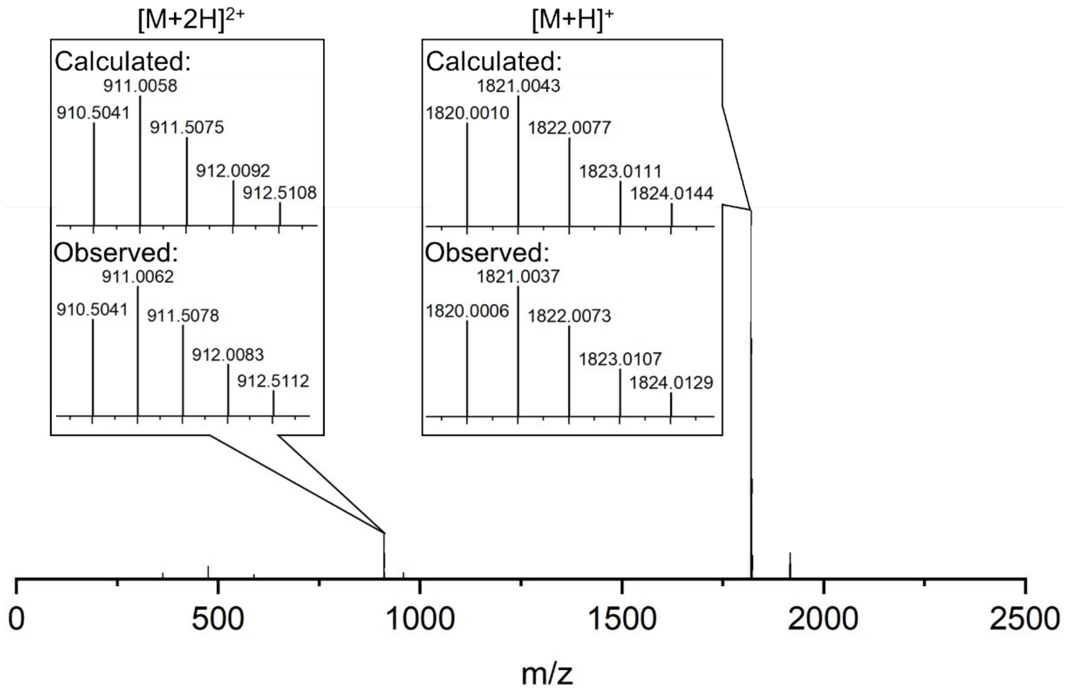

Fig. S21 ESI-HRMS of *R*-**4**.

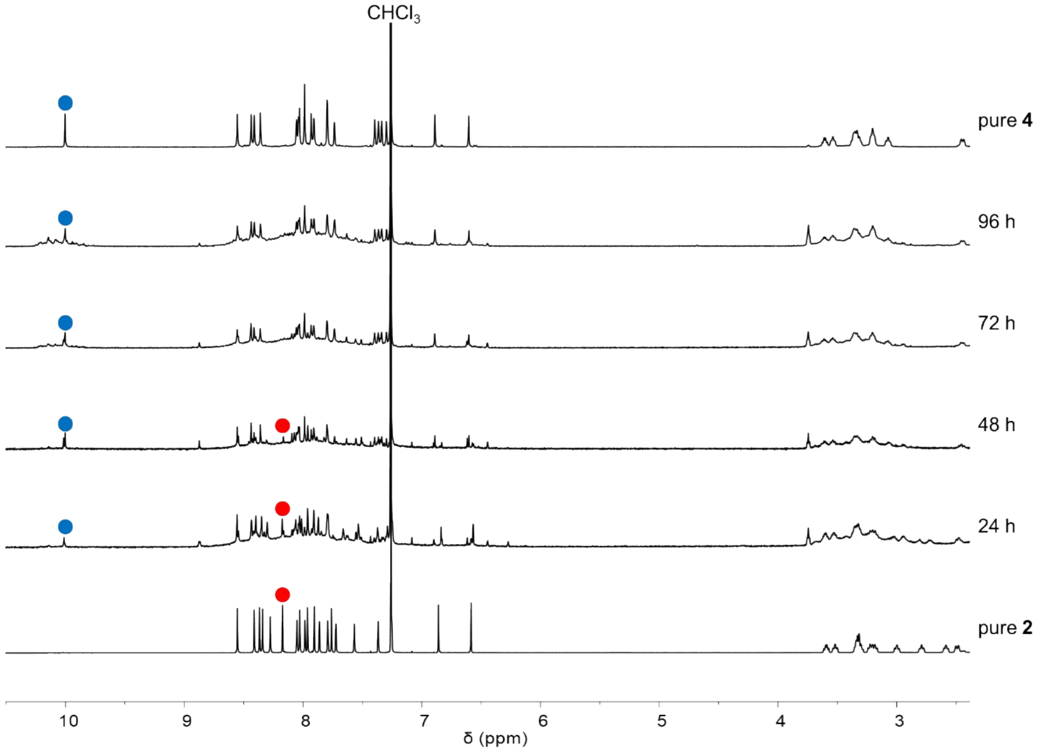

Fig. S22 Partial ^{1}H NMR spectra (600 MHz, $CDCl_3$, 298 K) of combining *R*-CHDA and excess **1**, which were recorded after certain amount of time. In the bottom and top showed the *R*-**2** and *R*-**4** in their pure form obtained *via* precipitation, respectively. The resonances labelled with blue and red circles correspond to the aldehyde protons in *R*-**4** and corresponding imine protons in *R*-**2**, respectively.

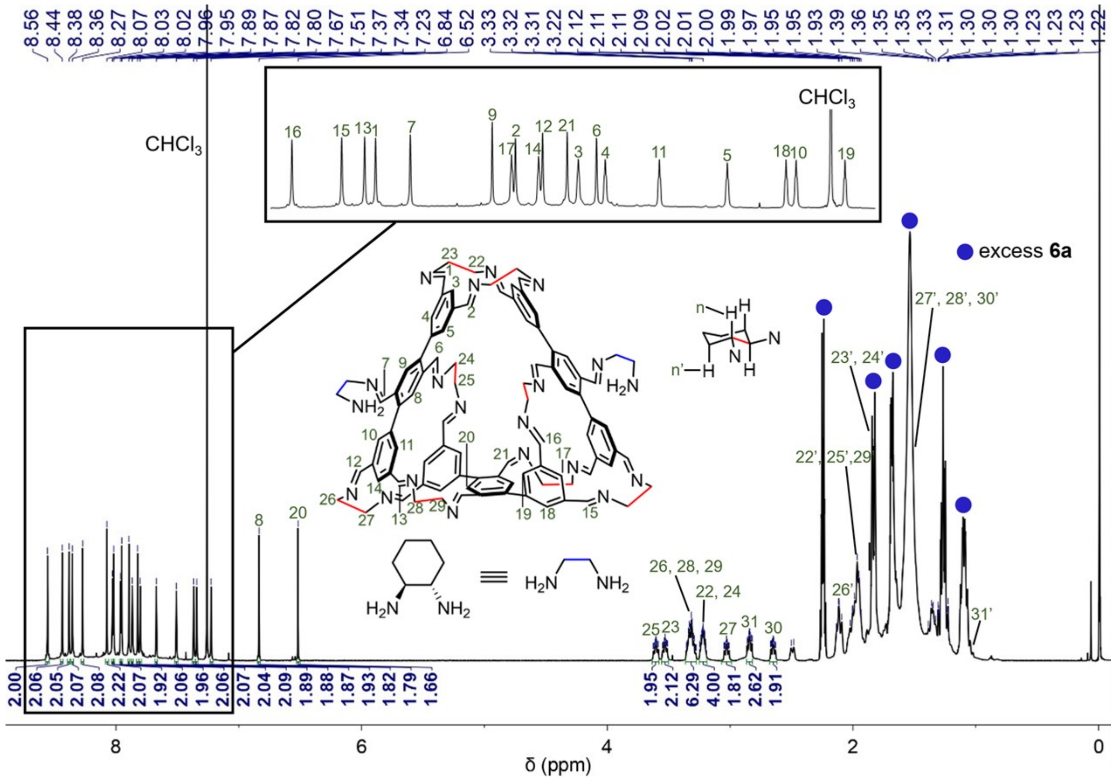

Fig. S23 Partial ^{1}H NMR spectrum (600 MHz, $CDCl_3$, 298 K) of *R*-**5a**. The resonances labelled with blue circles correspond to **6a**.

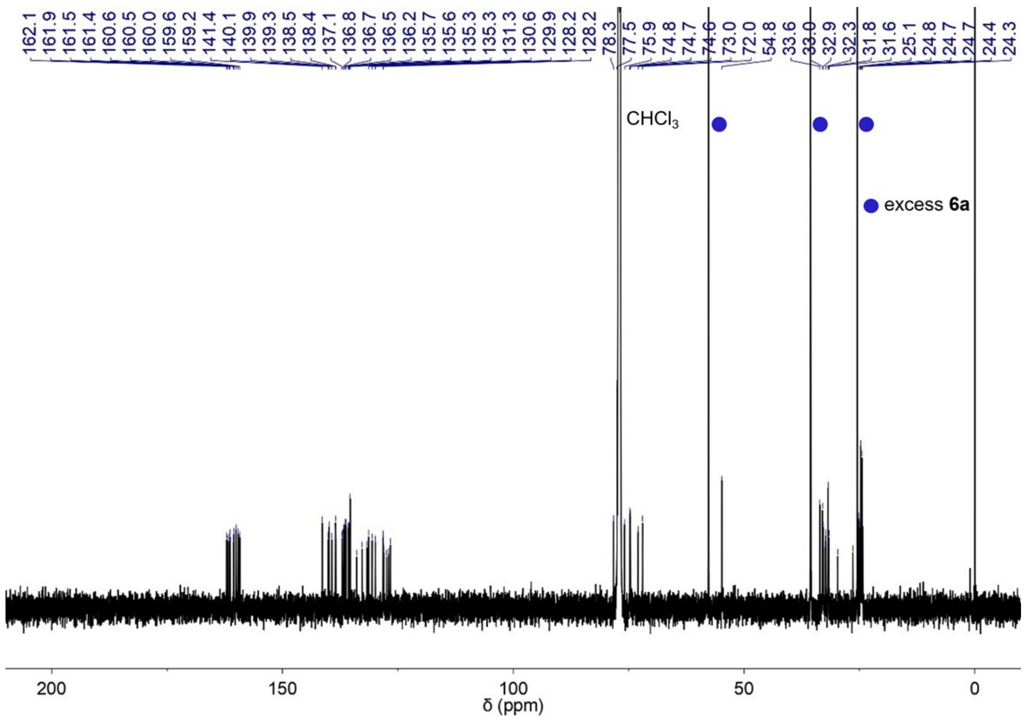

Fig. S24 Partial ^{13}C NMR spectrum (150 MHz, $CDCl_3$, 298 K) of *R*-**5a**. The resonances labelled with blue circles correspond to **6a**.

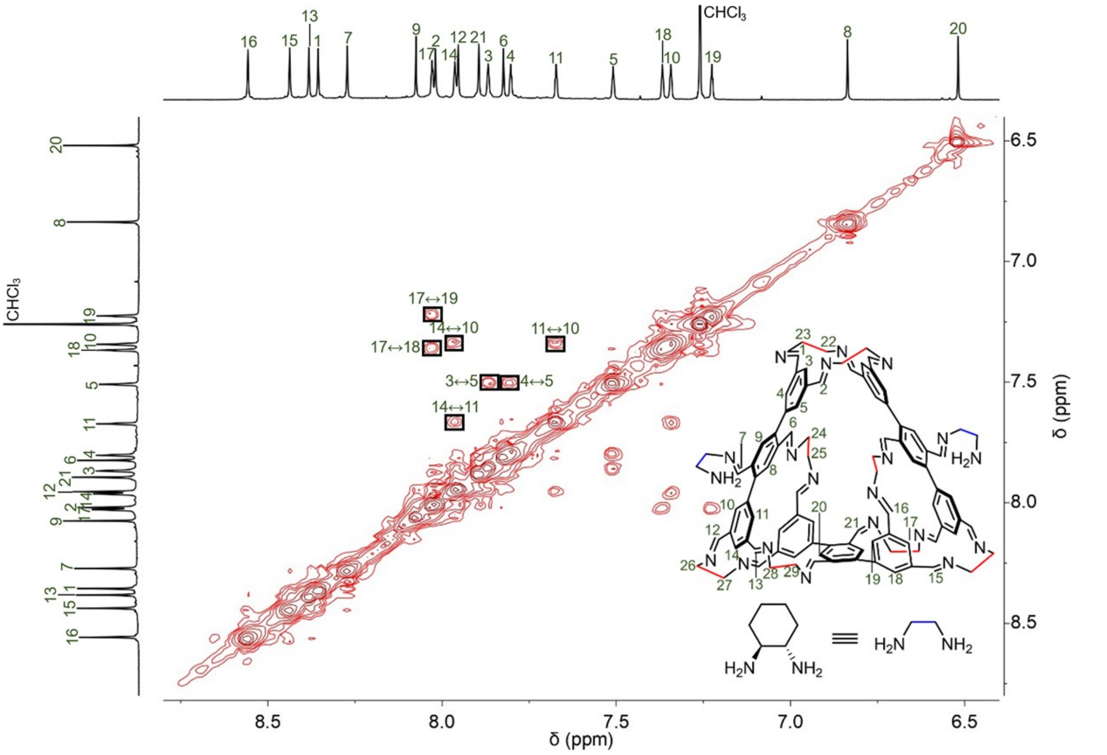

Fig. S25 Partial ^{1}H-^{1}H COSY NMR spectrum (600 MHz, $CDCl_3$, 298 K) of *R*-**5a**.

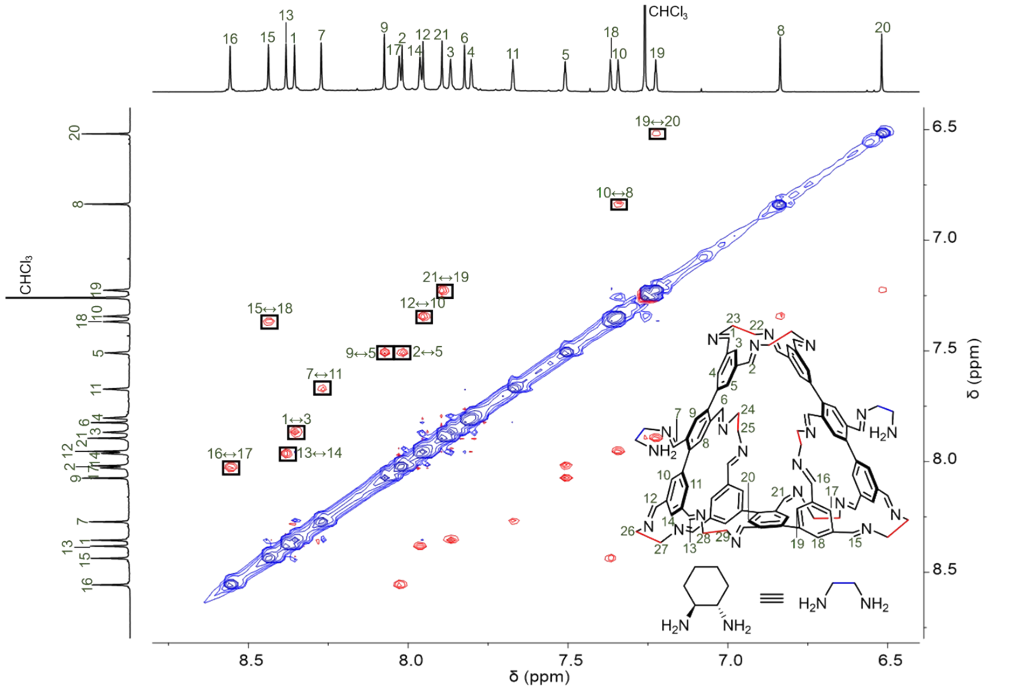

Fig. S26 Partial (Aromatic-aromatic region) ^{1}H-^{1}H ROESY NMR spectrum (600 MHz, $CDCl_3$, 298 K) of *R*-**5a**.

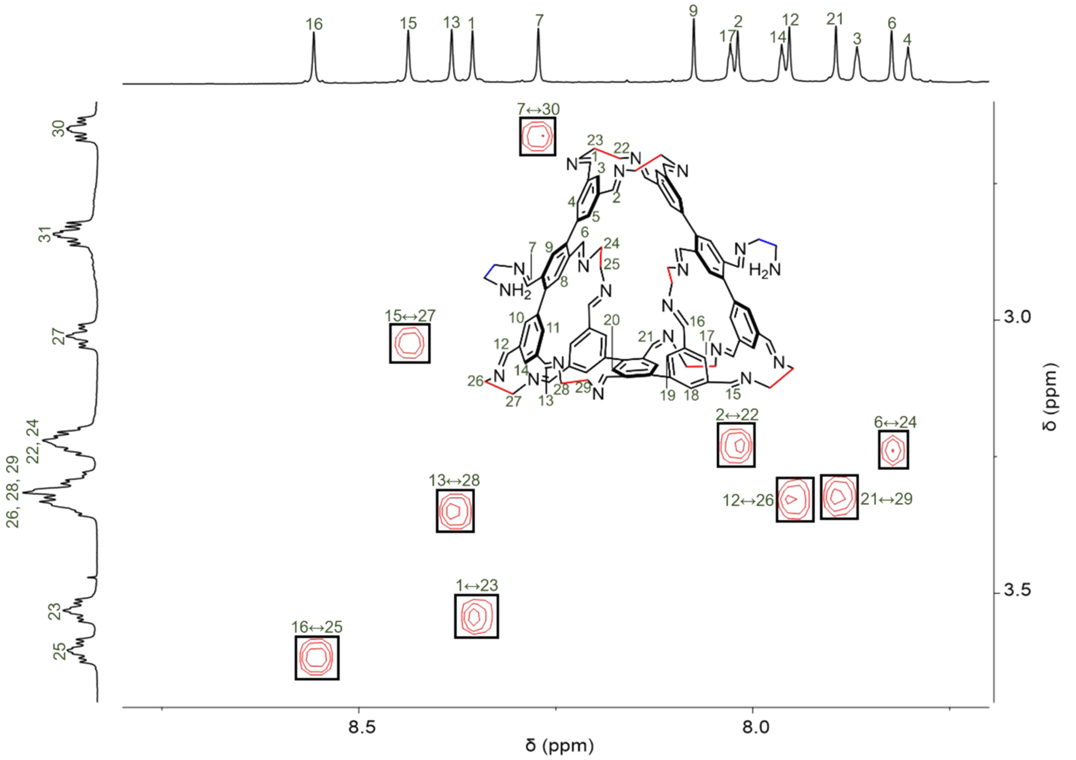

Fig. S27 Partial (Aromatic-aliphatic region)^{1}H-^{1}H ROESY NMR spectrum (600 MHz, $CDCl_3$, 298 K) of *R*-**5a**.

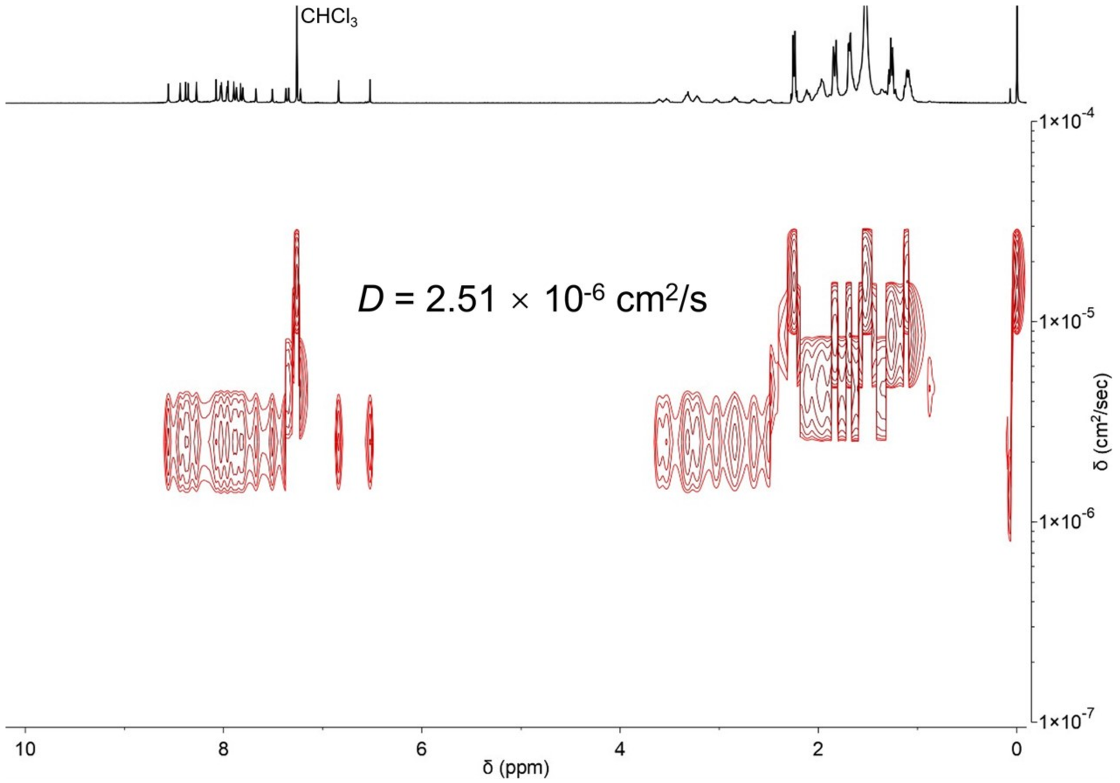

Fig. S28 DOSY spectrum (500 MHz, $CDCl_3$, 298 K) of *R*-**5a**. The diffusion coefficient (*D*) was determined to be 2.51×10^{-6} cm^2/s.

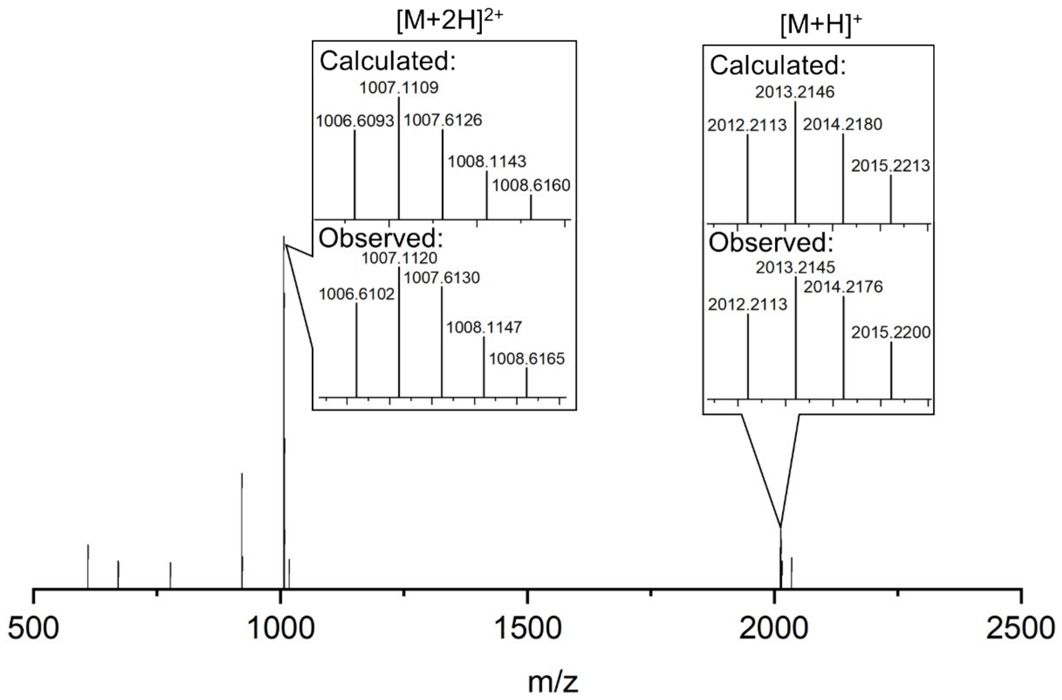

Fig. S29 ESI-HRMS of *R*-**5a**.

4. The Yields of *R*-**4** to *R*-**2** and *R*-**5**

The yields of the corresponding cages from *R*-**4** were determined by using 1,2-dichloroethane as the internal standard in the corresponding ^{1}H NMR samples of both the precursors and the corresponding reaction solution. The integration of the resonance of the internal standard namely dichloroethane was normalized to 5.00, allowing the concentration ratios of the [3+10] products relative to their corresponding [3+8] aldehyde precursors to be calculated. For example, in the ^{1}H NMR spectra of *R*-**4** and *R*-**2** (Figure S30), whose spectra were recorded before and after adding *R*-CHDA, the resonances of the protons in *20* position were integrated as 1.30 and 1.21, respectively, relative to the standard; the yield of *R*-**2** was thus calculated to be 93% (1.21/1.30). The yields mentioned below were all calculated *via* this method.

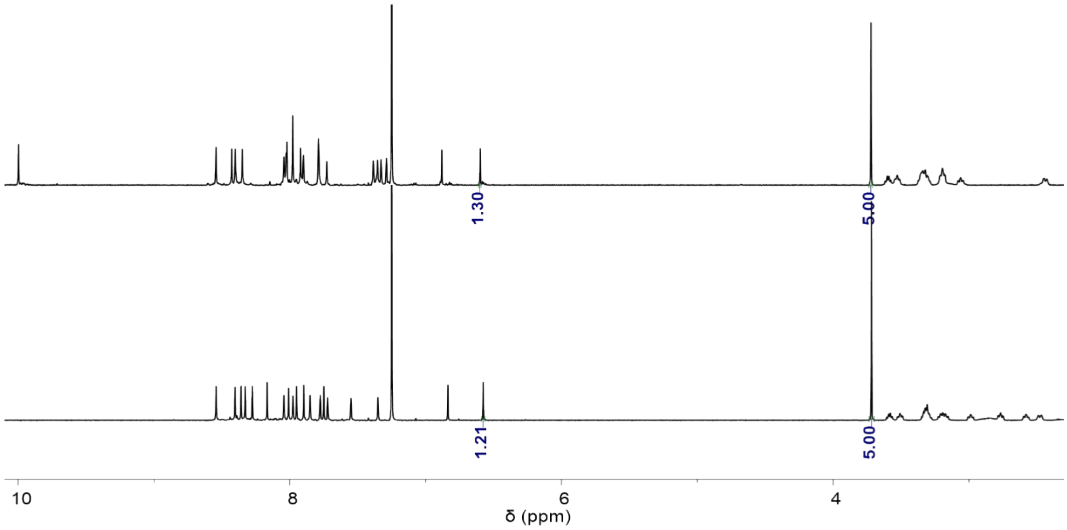

Fig. S30 Partial ^{1}H NMR spectrum (600 MHz, $CDCl_3$, 298 K) of *R*-**4** (top) and *R*-**2** (bottom).The NMR yield of *R*-**4** to *R*-**2** is 93%.

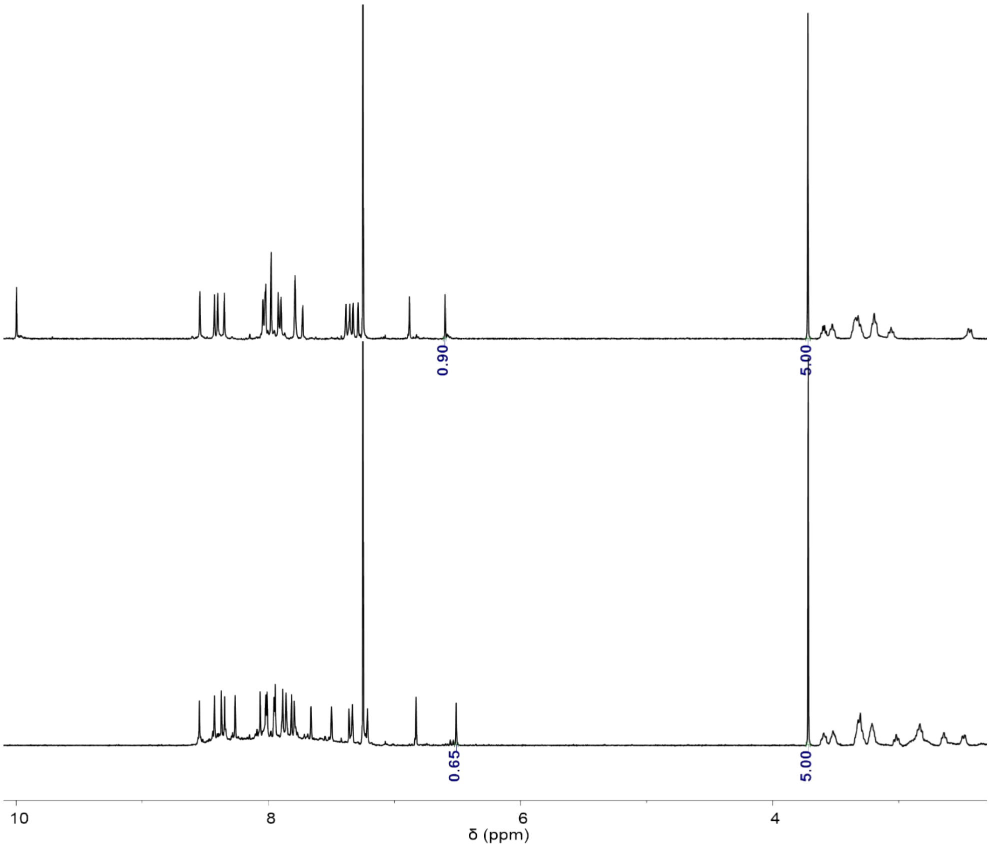

Fig. S31 Partial ^{1}H NMR spectrum (600 MHz, $CDCl_3$, 298 K) of *R*-**4** (top) and *R*-**5a** (bottom). The NMR yield of *R*-**4** to *R*-**5a** is 72%.

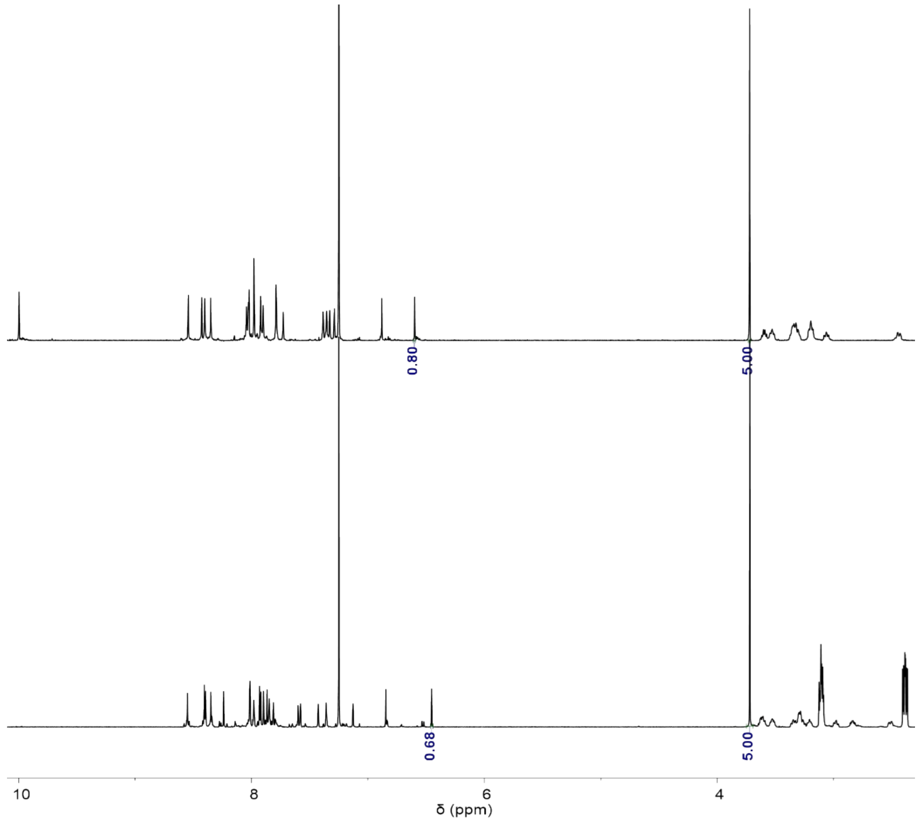

Fig. S32 Partial ^{1}H NMR spectrum (600 MHz, $CDCl_3$, 298 K) of *R*-**4** (top) and *R*-**5b** (bottom). The NMR yield of *R*-**4** to *R*-**5b** is 85%.

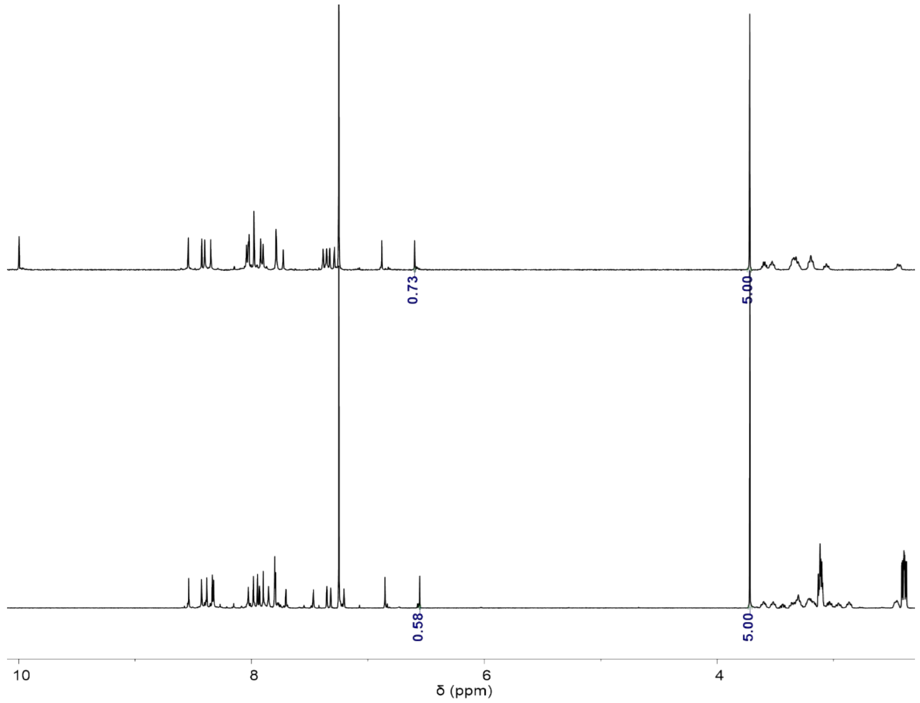

Fig. S33 Partial ^{1}H NMR spectrum (600 MHz, $CDCl_3$, 298 K) of *R*-**4** (top) and *R*-**5c** (bottom). The NMR yield of *R*-**4** to *R*-**5c** is 79%.

5. UV/Vis and Circular Dichroism Spectra

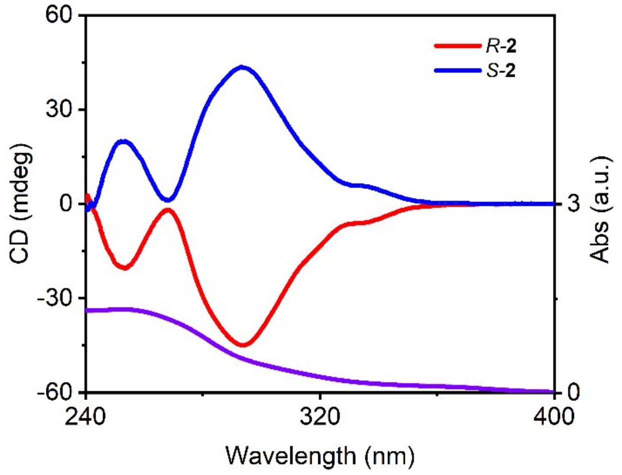

Fig. S34 UV/Vis (right) and CD (left) spectra (1.0×10^{-4} M, $CHCl_3$, 298K) of *R*-**2** and *S*-**2**.

6. X-ray Crystallography

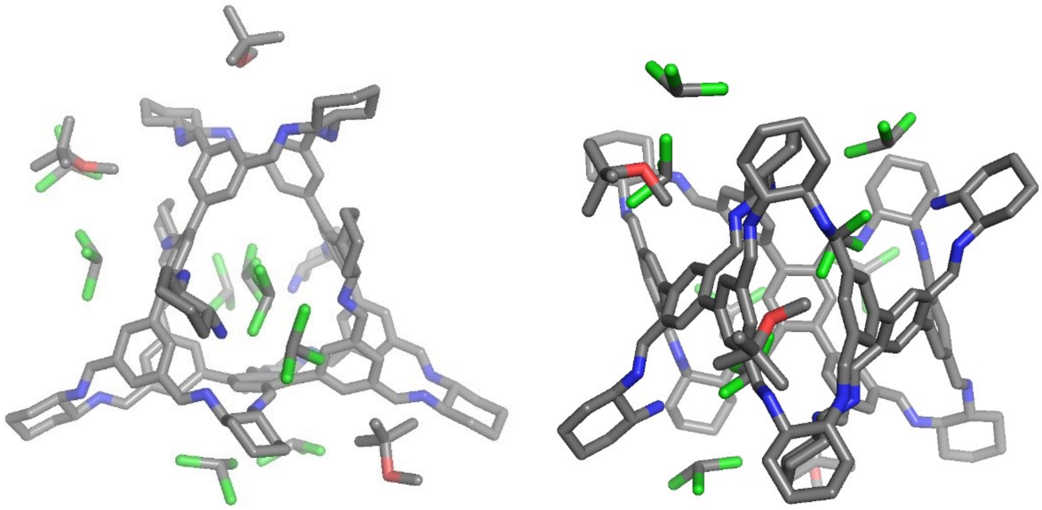

Fig. S35 Single-crystal X-ray diffraction structures of *R*-**2**: $C_{155}H_{190}Cl_{24}N_{20}O_3$, M = 3232.06, T = 100.00 K, monoclinic, space group $P2_1/n$, a = 21.0151(11) Å, b = 28.0832(15) Å, c = 28.4875(15) Å, α = 90°, β = 96.835(2)°, γ = 90°, V = 16693.0(15) Å^3, Z = 4. 102816 reflections collected, 24037 independent reflections. R(int) = 0.1602. Final R_1 (I>2σ(I)) = 0.1060 and wR_2 = 0.2685. CCDC number: 2446596.

6.1 Challenges in Crystal Growth and Selection.

The [3+10] non-closed structure **2** we synthesized exhibits a highly complex three-

dimensional architecture and incorporates two incompletely reacted CHDA units. These structural features presented significant challenges for the growth of single crystals. We systematically screened a variety of solvent systems, including good solvents such as chloroform, dichloromethane, dichloroethane, and tetrachloroethane, as well as anti-solvents like methyl tert-butyl ether, isopropyl ether, and diethyl ether. Despite extensive efforts, only a limited number of crystals suitable for X-ray analysis were obtained. Moreover, these crystals were generally limited in both size and diffraction quality.

6.2 Single-Crystal Data Collection

The synthesized single crystals were small, with approximate dimensions of 0.18 × 0.02 × 0.02 mm. As the structure consists mainly of light atoms (C, H, N, O), the inherent diffraction intensity was weak. Additionally, irreversible decomposition occured within seconds after removal from the mother liquor, further degrading diffraction quality. To address this, we used a more powerful liquid metal target source and ultra-low temperature conditions (100 K), with a scan step size of 1° and an exposure time of 3 s (standard exposure: 1 s). Over 1,400 diffraction images were collected across 6 runs, enabling structural solution. However, the issue of weak data was not fully resolved, so the data resolution was truncated at 0.9 Å, as reflections beyond this resolution exhibited a signal-to-noise ratio (S/N) < 2 even with extended exposure times.

X-ray single-crystal diffraction data were collected at 100K with a Bruker D8 Venture diffractometer metaljet with GaKα (λ = 1.34139 Å) for cell determination and subsequent data collection. Data reduction was performed by APEX5 software and multi-scan absorption correction was applied. Using Olex2 [1], the crystal structure was solved by ShelXT [2] and refined with full-matrix least-squares methods with anisotropic thermal parameters for all non-hydrogen atoms on F2 using SHELXL [3].

7. Theoretical Calculations

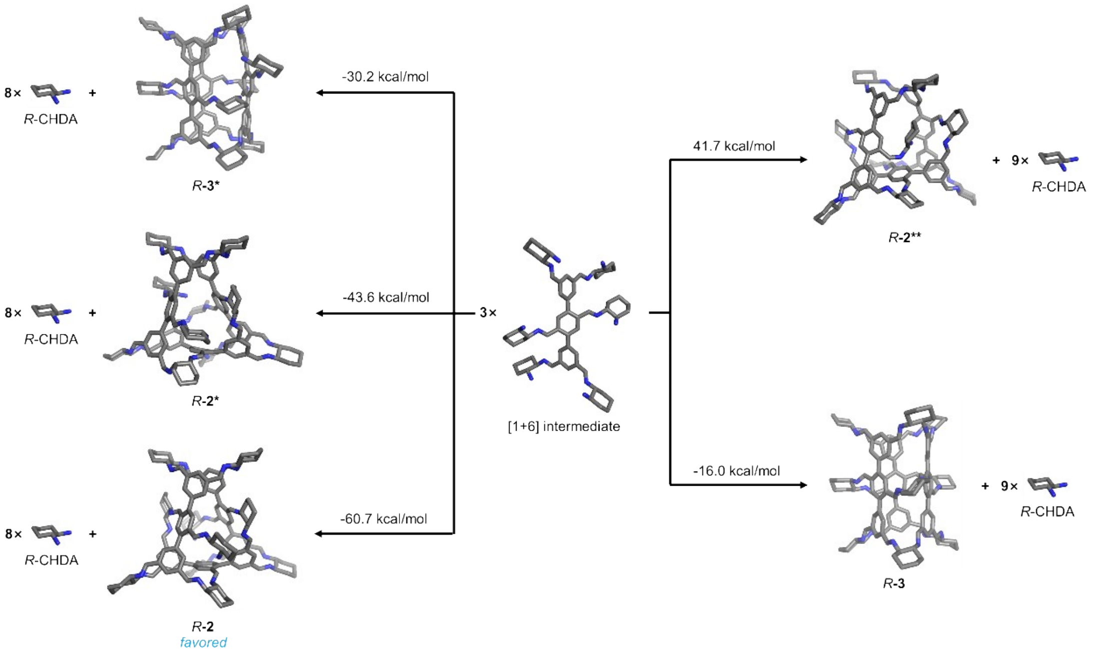

Fig. S36 The Gibbs free energy differences (ΔG) of the different optimized structures. Nitrogen, blue; Carbon, grey. Hydrogen atoms and irrelevant solvent molecules are removed for clarity.

The configurations of the [1+6] intermediate, *R*-**2**, *R*-**2***, *R*-**2****, *R*-**3*** and *R*-**3**** in the main text were optimized by using the density functional theory (DFT) at the M06-2X-D3/def2-TZVP/SMD(DCM)//B3LYP-D3BJ/6-31G*/IEFPCM(DCM) [4–10] level of theory with the Gaussian 16[11] package. Frequency analysis verified that all optimized geometries do not contain any imaginary frequencies. Gibbs free energies were corrected with the quasi-rigid-rotor-harmonic-oscillator (quasi-RRHO) [12] method.

In our time-dependent monitoring experiment, we observed that aldehyde precursor **1** initially forms a [1+6] intermediate, which subsequently converts into the [3+10] product. This pathway was confirmed by both ^{1}H NMR and mass spectrometry (Fig. S13 and S14), confirming that the transformation from this intermediate to the final products reflects the actual reaction pathway. Therefore, we calculated the Gibbs free energy changes for the process in which 3 equiv. of [1+6] intermediates connect in different ways while losing varying amounts of *R*-CHDA, yielding a series of possible structures (including three non-closed [3+10] structures and two closed [3+9] structures). The computational results demonstrated that when 3 equiv. of the [1+6] intermediate release 8 equiv. of *R*-CHDA to form the non-closed [3+10] structures *R*-

2, *R*-**2*** and *R*-**3***, the Gibbs free energy changes were -60.7 kcal/mol, -43.6 kcal/mol, and -30.2 kcal/mol, respectively. Conversely, releasing 9 equiv. of *R*-CHDA to generate the closed [3+9] structures *R*-**2**** and *R*-**3** yields Gibbs free energy changes of 41.7 kcal/mol and -16.0 kcal/mol, respectively. Thus, we can conclude that the formation of *R*-**2** through self-assembly is thermodynamically justified.

To provide readers with a clearer understanding of the preferential formation of *R*-**2** over *R*-**3**, we have explicitly calculated the Gibbs free energy changes for the following two processes (Fig. S37): Conversion of three equivalents of **1** and ten equivalents of *R*-CHDA into one equivalent of *R*-**2** and eighteen equivalents of water; conversion of three equivalents of **1** and nine equivalents of *R*-CHDA into one equivalent of *R*-**3** and eighteen equivalents of water.

In principle, the overall Gibbs free energy change for the complete reaction could be obtained by superimposing the Gibbs energy change for the formation of the [1+6] intermediate from aldehyde **1** and *R*-CHDA onto the pathway shown in Figure S36. Therefore, this comparative approach is equivalent to the original method based on Hess's Law.

The calculated values were -98.6 kcal/mol and -53.8 kcal/mol, respectively, with a difference of -44.8 kcal/mol. This confirms that the formation of the [3+10] structure *R*-**2** is more exothermic, which aligns with the original calculation starting from the [1+6] intermediate.

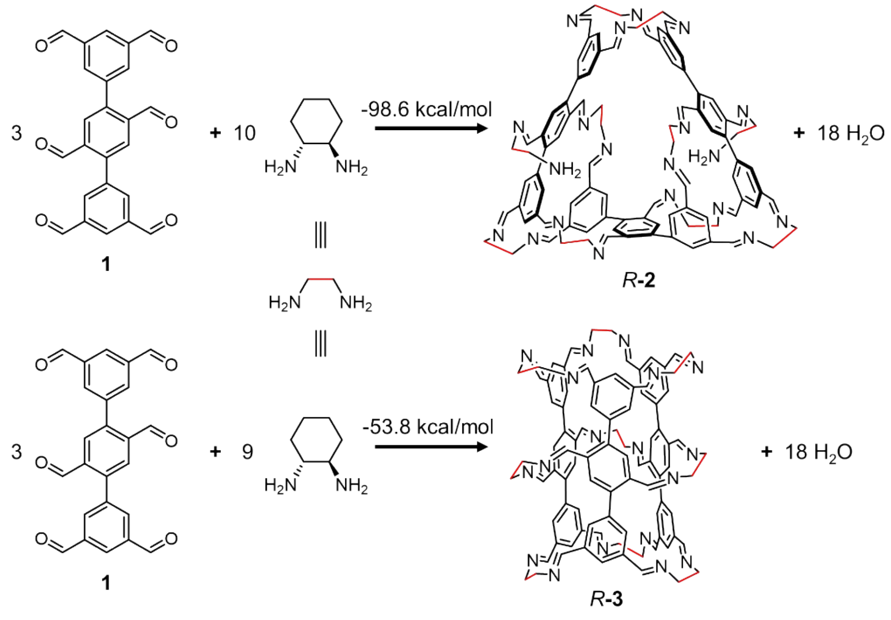

Fig. S37. The Gibbs free energy changes (ΔG) of the predicted (bottom) and observed (top) self-assembling reactions.

8. Reaction of *R*-**4** with Amines

Since *R*-**4** contains two aldehyde groups, we attempted to use it as a diformyl precursor to obtain more complex molecules. We thus added one of the following amines *R*-CHDA, *S*-CHDA (**6a**), (1*R*,2*R*)-2-aminocyclohexanol (**6b**), (1*S*,2*S*)-2-aminocyclohexanol (**6c**) or racemic *trans*-CHDA (**6d**) into the solution of *R*-**4**, followed by recording the corresponding ^{1}H NMR spectra. The corresponding solutions were stirred at room temperature for 24 h (Scheme S4). ^{1}H NMR spectra (Fig. S38) and ESI-MS (Fig. S39 and S40) showed that *R*-**4** reacted with two equivalents of each amine. For example, the ^{1}H NMR spectrum revealed that addition of *R*-CHDA to *R*-**4** afforded *R*-**2**. The usage of **6a**, **6b** and **6c** yielded products *R*-**5a**, *R*-**5b** and *R*-**5c** respectively. When racemic diamine **6d** was added, a more complex product mixture was obtained.

As a comparison, all of these products including *R*-**5a**, *R*-**5b** and *R*-**5c** were not obtained in one-pot manner. For example, combining **1**, *R*-CHDA and **6b** or **6c** produced complex, intractable mixtures (Fig. S42 and S43). This observation indicates that the successful formation of *R*-**5a**, *R*-**5b** and *R*-**5c** results from the kinetic stability of *R*-**4**.

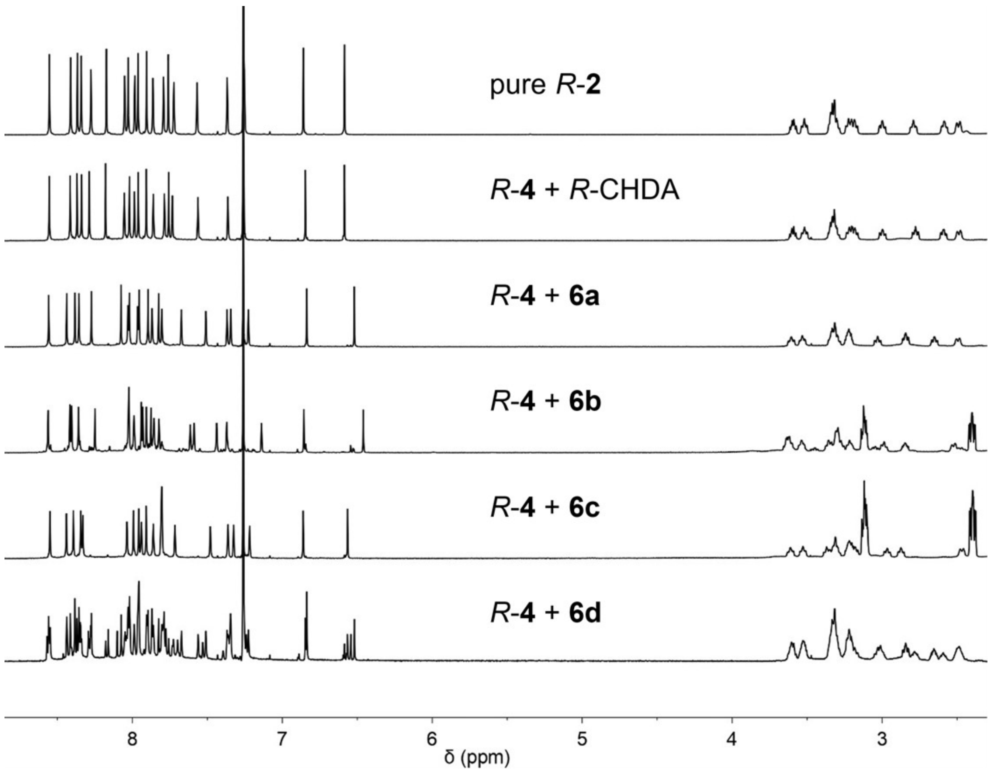

Fig. S38 Partial ^{1}H NMR spectra (600 MHz, $CDCl_3$, 298 K) of reaction of *R*-**4** with 10 e.q. of different amines (*R*-CHDA, **6a**, **6b**, **6c** and **6d**). In the top showed the ^{1}H NMR spectrum of *R*-**2** in their pure form.

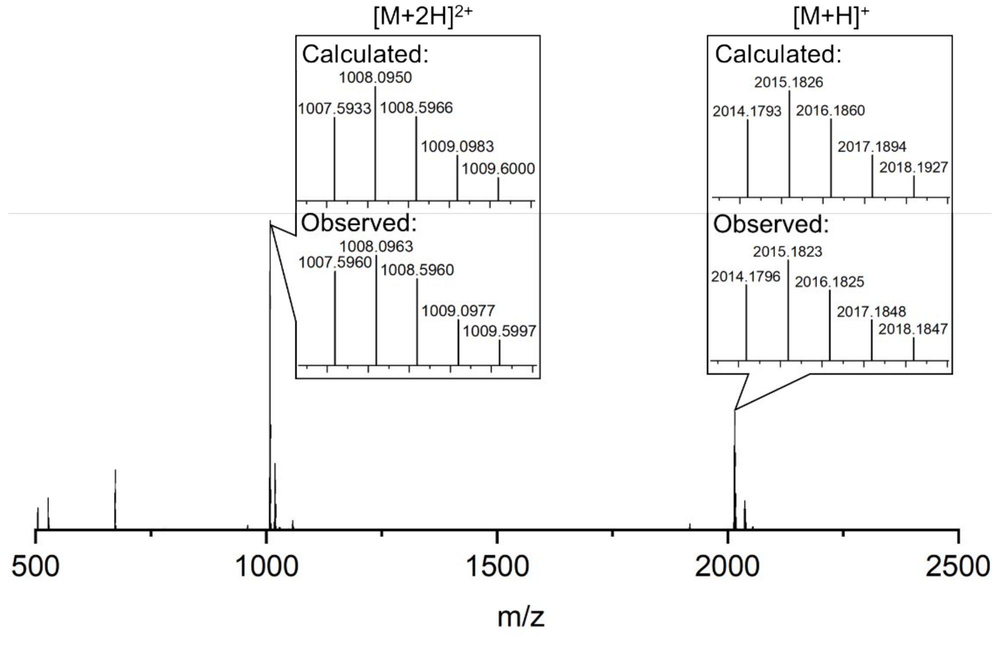

Fig. S39 ESI-HRMS of reaction solution of *R*-**4** and **6b**. *m/z* calculated for $C_{132}H_{146}N_{18}O_2^{2+}$($[M+2H]^{2+}$): 1007.5928; found: 1007.5960; *m/z* calculated for $C_{132}H_{145}N_{18}O_2^{+}$($[M+H]^{+}$): 2014.1792; found: 2014.1796.

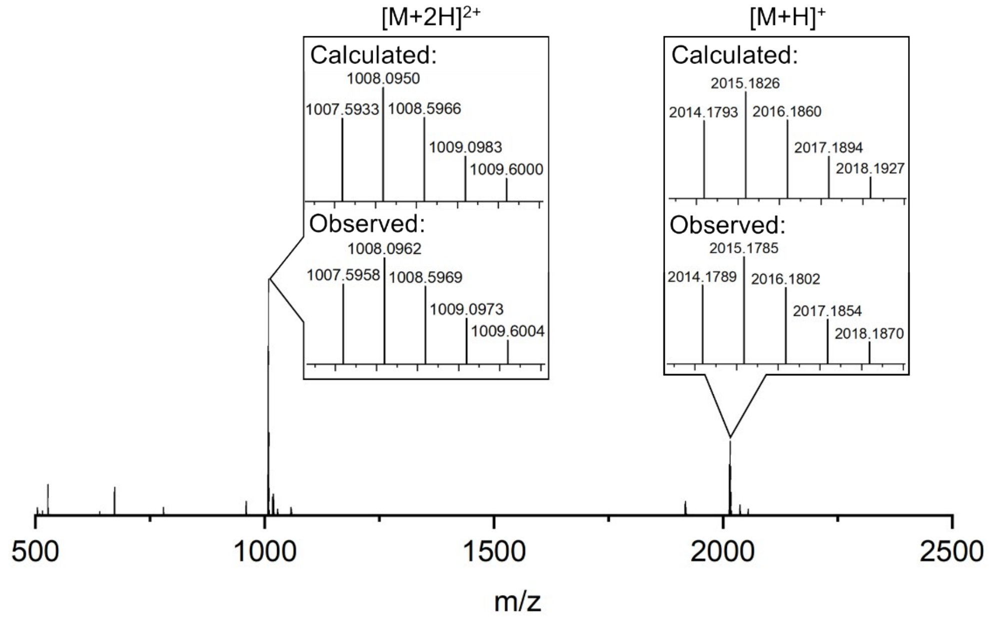

Fig. S40 ESI-HRMS of reaction solution of *R*-**4** and **6c**. *m/z* calculated for $C_{132}H_{146}N_{18}O_2^{2+}$([M+2H]$^{2+}$): 1007.5928; found: 1007.5958; *m/z* calculated for $C_{132}H_{145}N_{18}O_2^{+}$([M+H]$^{+}$): 2014.1792; found: 2014.1789.

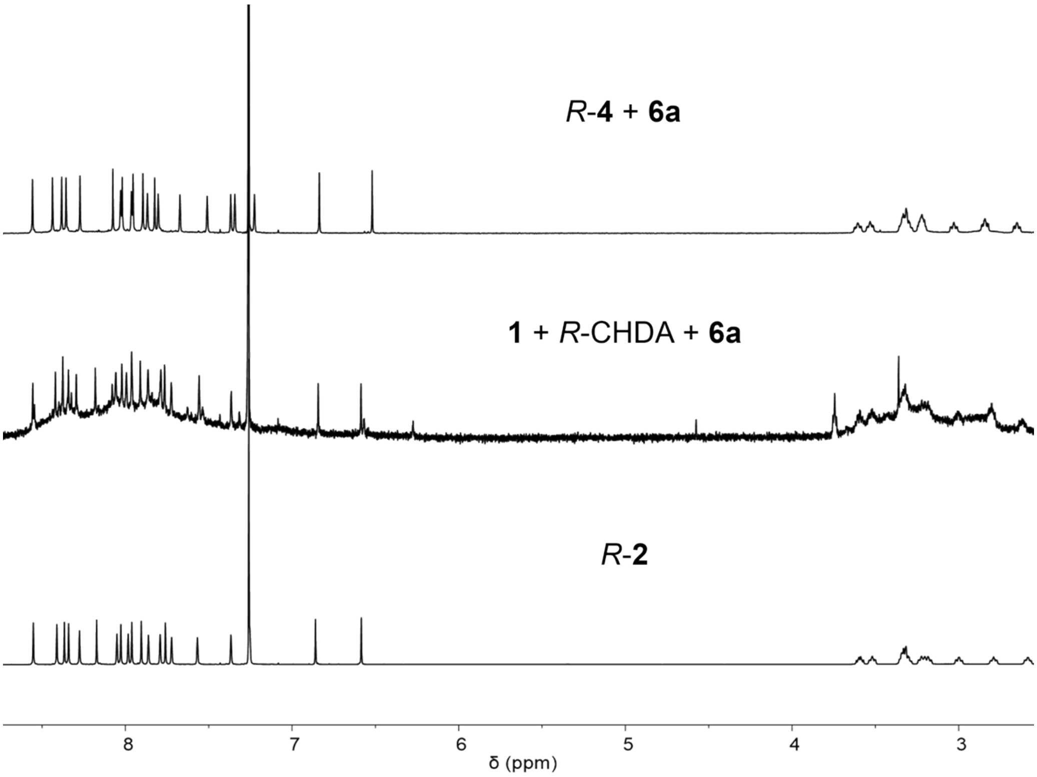

Fig. S41 Partial ^{1}H NMR spectrum (600 MHz, $CDCl_3$, 298 K) of *R*-**4** and **6a** (top), **1**, *R*-CHDA and **6a** (middle), *R*-**2** (bottom).

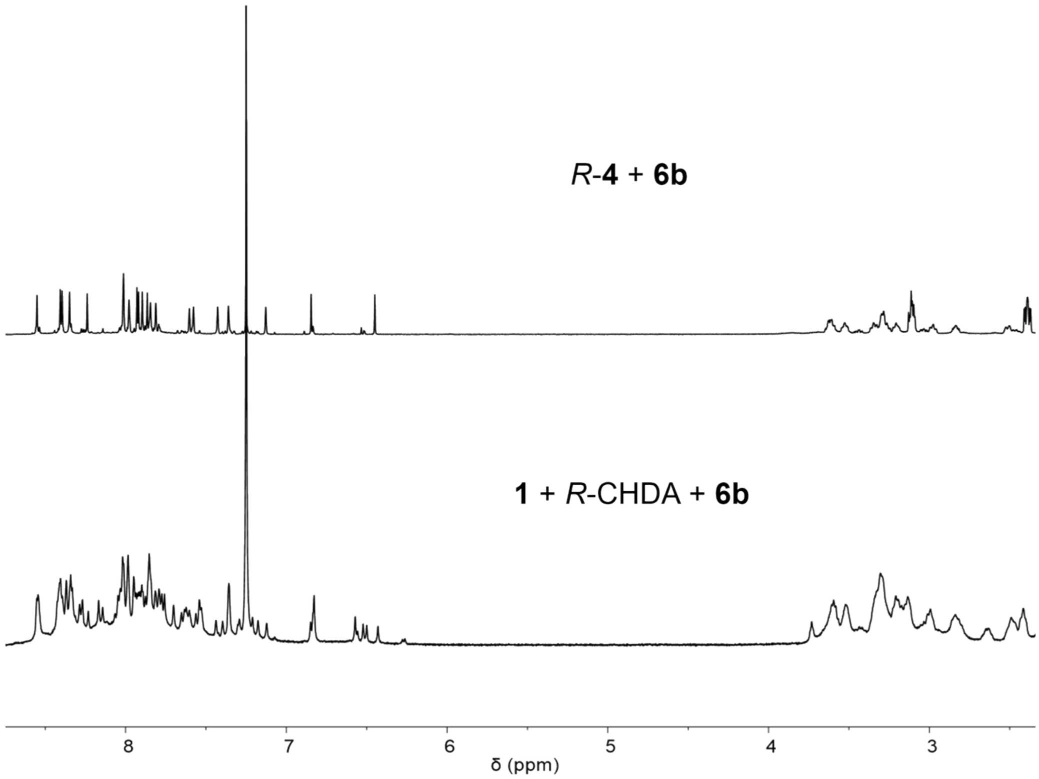

Fig. S42 Partial ^{1}H NMR spectrum (600 MHz, $CDCl_3$, 298 K) of *R*-**4** and **6b** (top), **1**, *R*-CHDA and **6b** (bottom).

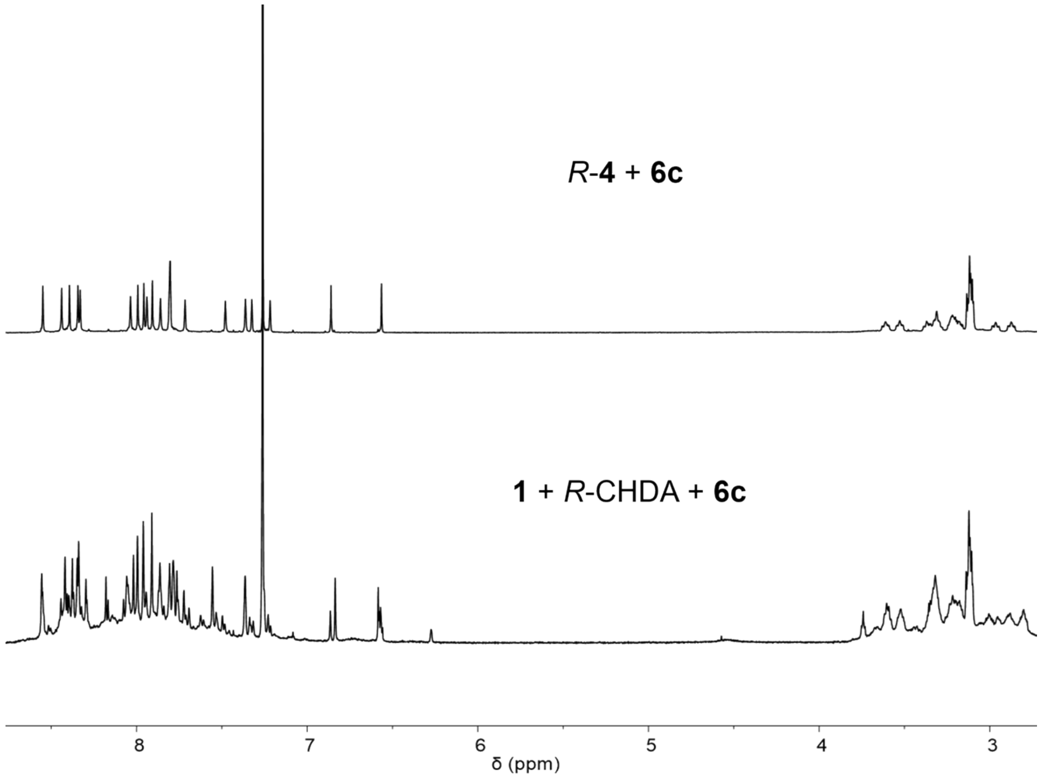

Fig. S43 Partial ^{1}H NMR spectrum (600 MHz, $CDCl_3$, 298 K) of *R*-**4** and **6c** (top), **1**, *R*-CHDA and **6c** (bottom).

9. Reference

1 O. V. Dolomanov, L. J. Bourhis, R. J. Gildea, J. A. K. Howard and H. Puschmanna, *J. Appl. Crystallogr.*, 2009, **42**, 339–341.

2 G. M. Sheldrick, *Acta Crystallogr., Sect. A: Found. Adv.*, 2015, **71**, 3–8.

3 G. M. Sheldrick, 2015, **71**, 3–8.

4 F. Weigend and R. Ahlrichs, *Phys. Chem . Chem . Phys.*, 2005, **7**, 3297–3305.

5 A. D. Becke, *Phys. Rev. A*, 1988, **38**, 3098–3100.

6 C. Lee, W. Yang and R. G. Parr, *Phys. Rev. B*, 1988, **37**, 785–789.

7 S. Grimme, S. Ehrlich and L. Goerigk, *J Comput Chem*, 2011, **32**, 1456–1465.

8 W. J. Hehre, R. Ditchfield and J. A. Pople, *J. Chem. Phys.*, 1972, **56**, 2257–2261.

9 Y. Zhao and D. G. Truhlar, *Theor Chem Account*, 2008, **120**, 215–241.

10 A. V. Marenich, C. J. Cramer and D. G. Truhlar, *J. Phys. Chem. B*, 2009, **113**, 6378–6396.

11 Gaussian 16 (version Revision A.01) Gaussian, Inc., Wallingford CT 2016.

12 S. Grimme, *Chemistry A European J*, 2012, **18**, 9955–9964.
